# Study on environmental factors affecting the quality of codonopsis radix based on MaxEnt model and all-in-one functional factor

**DOI:** 10.1038/s41598-023-46546-6

**Published:** 2023-11-25

**Authors:** Zixia Wang, Yanjun Jia, Pengpeng Li, Zhuoshi Tang, Yina Guo, Longxia Wen, Huaqiao Yu, Fang Cui, Fangdi Hu

**Affiliations:** 1 School of Pharmacy, Lanzhou University, Lanzhou, 730000 China; 2 State Key Laboratory of Applied Organic Chemistry, Lanzhou University, Lanzhou, 730000 China; 3 Codonopsis Radix Research Institute, Lanzhou, 730000 Gansu Province China; 4 Codonopsis Radix Industrial Technology Engineering Research Center, Lanzhou, 730000 Gansu Province China

**Keywords:** Plant sciences, Climate sciences, Ecology, Environmental sciences

## Abstract

Owing to the increasing market demand of Codonopsis Radix, the cropper blindly cultivates to expand planting area for economic benefits, which seriously affects the quality of Codonopsis Radix. Therefore, this study synthesized 207 batches of Codonopsis Radix and 115 ecological factors, and analyzed the suitable planting areas of *Codonopsis pilosula* under current and future climate change based on Geographic Information System (GIS) and MaxEnt model. Secondly, we evaluated the quality of Codonopsis Radix based on the all-in-one functional factor including chromatographic fingerprint, the index components, the effective compounds groups, the nutritional components, and the nutritional elements, and the quality regionalization of Codonopsis Radix was analyzed. Finally, the ecological factors affecting the accumulation of effective components of Codonopsis Radix were analyzed. This study found for the first time that the highly suitable area of *Codonopsis pilosula* was mainly distributed in the Weihe River system and the Bailongjiang River system in Gansu Province. There were differences in the quality of Codonopsis Radix from different ecologically suitable areas based on the all-in-one functional factors, and the comprehensive high-quality area of Codonopsis Radix was mainly distributed in Longnan and Longxi district of Gansu Province. The precipitation, temperature and altitude play a key role in the accumulation of chemical components in the 10 ecological factors affecting the distribution of *Codonopsis pilosula*. Under future climatic conditions, the highly suitable area of *Codonopsis pilosula* is decreased.

## Introduction

Codonopsis Radix is a tonic TCM that contains polysaccharides, oligosaccharides, sesquiterpenes, triterpenoids, and other medicinal chemical ingredients^1–4^ and is rich in amino acids, proteins, K, Mg, Ca, Fe, Na, and other nutrients^5–7^. It has been used in food and traditional folk medicine prescriptions for thousands of years in China, Japan, South Korea, and other Asian countries^8^. At present, it plays an important role in the clinical preparation and compatibility of TCM and is also an important health food popular in China and Southeast Asia for strengthening the spleen and benefiting the lungs and immune regulation^9^. *C. pilosula*, *C. pilosula* var. *modesta*, and *C. tangshen* are the three varieties of Codonopsis Radix in Chinese Pharmacopoeia. Although Codonopsis Radix is widely distributed in China, the main producing areas of the three varieties of Codonopsis Radix are different. *C. pilosula* is mainly distributed in Shanxi and Longxi of Gansu, *C. pilosula* var. *modesta* is only distributed in the south of Gansu Province, and *C. tangshen* is mainly distributed in Sichuan and Chongqing^10^. In recent years, with the increase in the demand for medicinal and edible Codonopsis Radix, to seek economic benefits and expand the planting area of *Codonopsis pilosula*, under the guidance of reasonable planting, different varieties of *Codonopsis pilosula* were blindly planted in disorder, resulting in different varieties of *Codonopsis pilosula* failed to grow in suitable areas^11^. The effective components of medicinal plants are the material basis for the efficacy of TCM. Most of them are secondary metabolites of plants, which are greatly affected by environmental factors, and have time specificity and space specificity. The chaotic planting pattern seriously affects the quality of Codonopsis Radix^12,13^. Therefore, it is important to establish an effective analysis method to excavate suitable areas for the cultivation of *Codonopsis pilosula*, to improve the quality of Codonopsis Radix.

In recent years, global climate change has been obvious, and extreme weather has increased. People have realized the potential global climate change problem. The United Nations Intergovernmental Panel on climate change (IPCC) simulates the future climate change status under different emission levels through various climate models. Among them, SSP126 and SSP585 in the Shared socioeconomic Pathways (SSP)^14,15^ represent the climate conditions when the global temperature increases by 1.8 °C and 4.4 °C, respectively, by the end of this century^16^, and these climate scenarios can well explain future climate changes under different development conditions. Many studies have found that climate change has a far-reaching impact on the distribution range of species, and their suitable range will shrink or move significantly in response to expected climate change^17,18^. Predicting the extent of future shifts in suitable habitat for *Codonopsis pilosula* and how to cope with changes in climatic conditions is important for the planning and sustainability of *Codonopsis pilosula* cultivation. Currently, ecological niche modeling (ENM) analysis methods are favorable tools for predicting the possible distribution regions of species. The maximum entropy theory (MaxEnt) model has the advantages of short elapsed time, high modeling accuracy, and is the best ENM for prediction^19^. The MaxEnt model has been broadly applied to predict the potential distribution regions of medicinal plants^20,21^, and then with the spatial analysis function of the Geographic Information System (GIS) platform, the spatial quantification of TCM quality can be realized^22^. In recent years, more and more studies have combined MaxEnt models with GIS to analyze the habitat suitability of Chinese herbs^23,24^. For example, a study combined GIS and MaxEnt model to analyze the ecological suitability distribution of *Codonopsis pilosula* in Dingxi City, Gansu Province, China, indicating that the main ecological factors affecting the ecological suitability of *Codonopsis pilosula* are altitude, precipitation in April, etc^25^. Another study carried out the zoning and evaluation of the suitability of *Codonopsis pilosula* in China based on GIS and MaxEnt model, and combined with the 148 *Codonopsis pilosula* distribution information collected, the results showed that there were spatial differences of the suitable planting areas of the three varieties of *Codonopsis pilosula*^26^. The above study provides a constructive reference for the division of suitable areas for *Codonopsis pilosula*. However, *Codonopsis pilosula* in Gansu Province is not only distributed in Dingxi City, Longnan City is also the main producing area of *Codonopsis pilosula* in Gansu Province. In addition, the study of the suitability of *Codonopsis pilosula* in China requires a large amount of data on the distribution of *Codonopsis pilosula* to elucidate the suitability zone of *Codonopsis pilosula*, and the collected data have great limitations. Several studies have been conducted based on the MaxEnt model to explore the habitat of other major medicinal plants in Gansu Province, *Angelica sinensis* (Oliv.) Diels^27^ and *Cistanche deserticola* Ma^28^ and they all showed good results. The ecological suitability analysis of *Codonopsis pilosula* based on large samples and main producing areas in Gansu Province is important for the rational cultivation of *Codonopsis pilosula* and lays the foundation for the study of the quality zoning of *Codonopsis pilosula* (Table 1).

**Table 1:** Ecological factors variable information.

Abbreviation	Ecological factors	Unit
bio-1	Annual Mean Temperature	°C
bio-2	Mean Diurnal Range	°C
bio-3	Isothermality	1
bio-4	Temperature Seasonality	1
bio-5	Max Temperature of Warmest Month	°C
bio-6	Min Temperature of Coldest Month	°C
bio-7	Temperature Annual Range	°C
bio-8	Mean Temperature of Wettest Quarter	°C
bio-9	Mean Temperature of Driest Quarter	°C
bio-10	Mean Temperature of Warmest Quarter	°C
bio-11	Mean Temperature of Coldest Quarter	°C
bio-12	Annual Precipitation	mm
bio-13	Precipitation of Wettest Month	mm
bio-14	Precipitation of Driest Month	mm
bio-15	Precipitation Seasonality	mm
bio-16	Precipitation of Wettest Quarter	mm
bio-17	Precipitation of Driest Quarter	mm
bio-18	Precipitation of Warmest Quarter	mm
bio-19	Precipitation of Coldest Quarter	mm
prec(1–12)	Precipitation	mm
tavg(1–12)	Mean Temperature	°C
tmax(1–12	Maximum Temperature	°C
tmin(1–12)	Minimum Temperature	°C
vapr(1–12)	Water Vapor Pressure	kPa
wind(1–12)	Wind Speed	m·s^-1^
srad(1–12)	Solar Radiation	kJ·m^-2^·day^-1^
*t*_pH	Topsoil pH	1
*t*_cec_soil	Topsoil CEC	cmol·kg^-1^
*t*_sand	Topsoil sand fraction	%
*t*_clay	Topsoil clay fraction	%
*t*_oc	Topsoil organic carbon	%
su_sym90	Soil Unit Symbol (FAO-90)	1
awc_class	AWC Range	1
*t*_usda_tex_class	Topsoil USDA Texture Classification	1
elev	Altitude	m
slope	Slope	°
aspect	Aspect	1
zblx	Vegetation Type	1

In the current studies based on ecological factors to evaluate the quality of Codonopsis Radix, the selection of chemical components is mostly focused on their single component or chromatographic fingerprint. For example, Wu^29^ evaluated the influence of ecological factors on the quality of Codonopsis Radix*.* Based on the chromatographic fingerprint of Codonopsis Radix. Wan et al^30^. evaluated the impact of ecological factors on the quality of Codonopsis Radix in Dingxi district by establishing a relevant model between ecological factors and common peaks. In a sense, it is more meaningful to establish a chromatographic fingerprint to divide the suitability area than to use the concentration of a pharmacodynamic component to complete the analysis. However, in the chromatographic fingerprint that was constructed by using ethanol or methanol solvent extracts, it had been impossible to achieve scientific characterization of the polysaccharides^31^, amino acids, and proteins^32^ of Codonopsis Radix, but the above components just showed significant efficacy and activity. Our previous study showed that carbohydrate components are the main components of its efficacy, polysaccharides have antitumor activity^33^, and oligosaccharides have immunomodulatory effects^34^. Meanwhile, Codonopsis Radix is rich in nourishing protein, amino acids, and other nutrients^5,6^. Some studies have found that Codonopsis Radix is rich in nutrient elements, such as Fe, Na, K, Ca, Mg, Zn, Sr^7^, and these elements play an essential role in sustaining bone and muscle power^35^, preventing and treating metabolic diseases^36^, and preventing cancer^37^. In addition, lobetyolin^38^, atractylenolide III^39^, syringin^40^, and alcohol extract^41^ have also been shown to have significant pharmacological effects. And we have confirmed that comprehensive evaluation using the above components as indicators can significantly distinguish Codonopsis Radix with different growth years^6^. Therefore, the comprehensive evaluation of the quality zoning of Codonopsis Radix needs to be considered both from the perspective of medicinal and nutritional components. Based on the chromatographic fingerprint, the carbohydrate components accounting for 3/4 of the total extract of Codonopsis Radix and other medicinal and nutritional components were integrated to construct the all-in-one functional factors of Codonopsis Radix.

In this study, the MaxEnt model and GIS technology were combined to study the potential habitat suitability areas of *Codonopsis pilosula* under different climate scenarios in the present and future. Further, functional factors such as the common peaks in fingerprint, the index components, the effective compound groups, the nutritional components, and the nutritional elements were first used as indicators to carry out the quality evaluation of Codonopsis Radix, and the influence of climatic factors on the quality of Codonopsis Radix was analyzed by the all-in-one functional factor. The results can provide a reference basis for the rational planning of *Codonopsis pilosula* cultivation areas in Gansu Province and are important for producing stable quality Codonopsis Radix and maximizing its resource value.

## Results

### Habitat suitability analysis of *Codonopsis pilosula*

**Figure 1:**
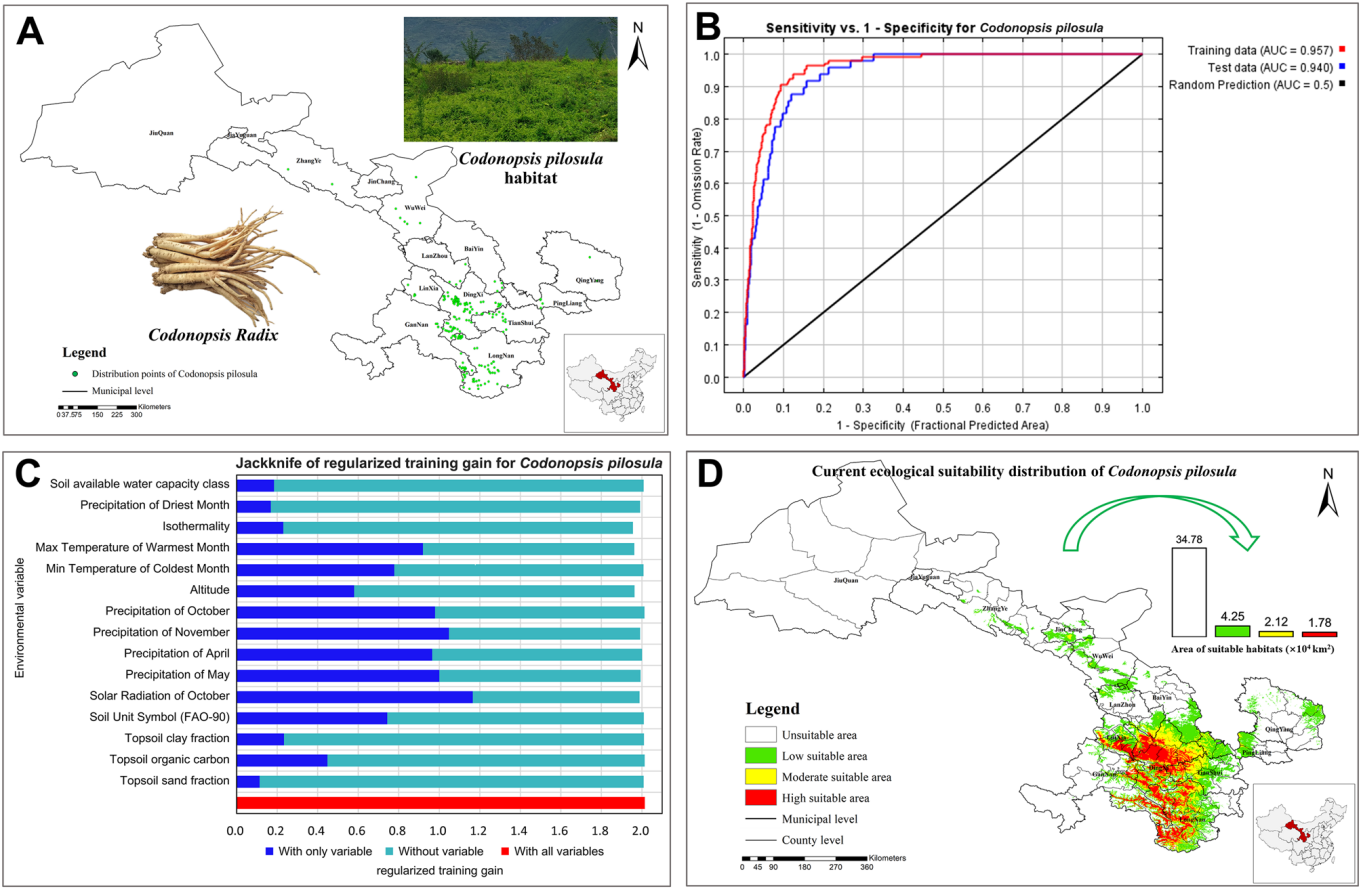
(**A**) Spatial distribution of *Codonopsis pilosula* in Gansu Province, (**B**) The ROC curves of MaxEnt models for *Codonopsis pilosula*, (**C**) The results of the jackknife test are of variable importance, (**D**) The habitat suitability distribution map of *Codonopsis pilosula* in Gansu Province (The maps were prepared by Zixia Wang and Yanjun Jia in ArcGIS Pro, https://www.esri.com/zh-cn/arcgis/products/arcgiscn/arcgis/products/arcgis-pro/resources).

Download the 1:4,000,000 administrative map of China from the National Geographic Information Data website (http://nfgis.nsdi.gov.cn/), and processed it through ArcGis software to obtain the administrative map of Gansu Province. Based on the collected longitude, and latitude of each occurrence point, combined with the administrative map vector data of Gansu Province to generate the spatial distribution map of *Codonopsis pilosula* in Gansu Province through ArcGis (Fig. 1A). The output results of the accuracy test of the *Codonopsis pilosula* maximum entropy model is displayed in Fig. 1B and Fig. S1. The omission rate of training samples in the analysis omission/debugging curve was consistent with the prediction omission rate, the AUC values of the training set and the test set in the receiver operating curve were 0.957 and 0.940, respectively. Indicating the prediction effect of the model was excellent. In conclusion, the modeling results can be used for habitat suitability analysis of *Codonopsis pilosula*.

Through multiple iterative arithmetics on 115 ecological factors, excluded those with a contribution rate of 0, and then through correlation analysis, 15 important ecological factors were obtained. Their percent contribution and importance are shown in Table 2. The contribution of ecological factors to the distribution of *Codonopsis pilosula* was determined according to the results of the Jackknife method. In the bar chart, the longer the blue one and the shorter the green one indicating that the represent variable has a more important effect on the distribution of *Codonopsis pilosula.* According to the results (Fig. 1C), the weight order of each ecological factor on the distribution of *Codonopsis pilosula* was as follows: Solar Radiation of October (srad-10), Precipitation of November (prec-11), Precipitation of May (prec-5), Precipitation of October (prec-10), Precipitation of April (prec-4), Max Temperature of Warmest Month (bio-5), Min Temperature of Coldest Month (bio-6), Soil Unit Symbol (FAO-90) (su_sym90), Altitude (elev), Topsoil Organic Carbon (*t*_oc), Topsoil Clay Fraction (*t*_clay), Isothermality (bio-3), Soil available water capacity class (awc_class), Precipitation of Driest Month (bio-14), Topsoil Sand Fraction (*t*_sand). According to the percent contribution, permutation importance, and weight, the common ecological factors were taken as the main ecological factors, and the cumulative contribution rate reached 92.7%. To sum up, precipitation, temperature, altitude, and solar radiation have the greatest effect on the growth of *Codonopsis pilosula*. Combined with the results of the knife-cut test and the contribution rate, the univariate response curve analysis was performed on the 10 main ecological factors affecting the distribution of *Codonopsis pilosula*. When the probability value was greater than 0.30, the variable range was more suitable for species distribution. When the probability value was greater than 0.50, the variable range was most suitable for species distribution. The results are shown in Table S1 and Fig. S2.

**Table 2:** Percentage contribution and permutation importance of ecological factors.

Ecological factors	Percent contribution/%	Permutation importance/%
Precipitation of October	37.3	1.4
Solar Radiation of October	20.1	3.2
Precipitation of November	9.9	4.6
Max Temperature of Warmest Month	7.1	20.9
Soil Unit Symbol (FAO-90)	5.7	0.6
Isothermality	5.7	1.6
Precipitation of May	4.4	5.7
Soil available water capacity class	3.5	1
Precipitation of Driest Month	2.2	0.7
Altitude	2.1	18.4
Topsoil organic carbon	0.9	0.2
Precipitation of April	0.4	39.7
Min Temperature of Coldest Month	0.3	1.4
Topsoil sand fraction	0.2	0.4
Topsoil clay fraction	0.2	0.2

The habitat suitability results of *Codonopsis pilosula* calculated by MaxEnt model software were imported into ArcGIS software to obtain the habitat suitability distribution map of *Codonopsis pilosula* in Gansu Province, and the potentially suitable area of *Codonopsis pilosula* was calculated (Fig. 1D and Table S3). The suitability distribution area of *Codonopsis pilosula* was mainly located in the southeast of Gansu Province. The total area of highly suitable area was 1.78 × 10^4^ km^2^, accounting for 4.15% of the area in Gansu Province, mainly distributed in Dingxi City and Longnan City. There was also a small amount of distribution in Linxia Hui Autonomous Prefecture, Gannan Tibetan Autonomous Prefecture, and Tianshui city. The moderate suitable area and low suitable area areas for *Codonopsis pilosula* were 2.12 × 10^4^ km^2^ and 4.25 × 10^4^ km^2^, respectively, accounting for 4.94% and 9.90% of the total area of Gansu Province, moderate suitable area mainly distributed around the highly suitable area, and low suitable area was distributed in other areas of Gansu Province except for Jiuquan City and Jiayuguan City.

**Figure 2:**
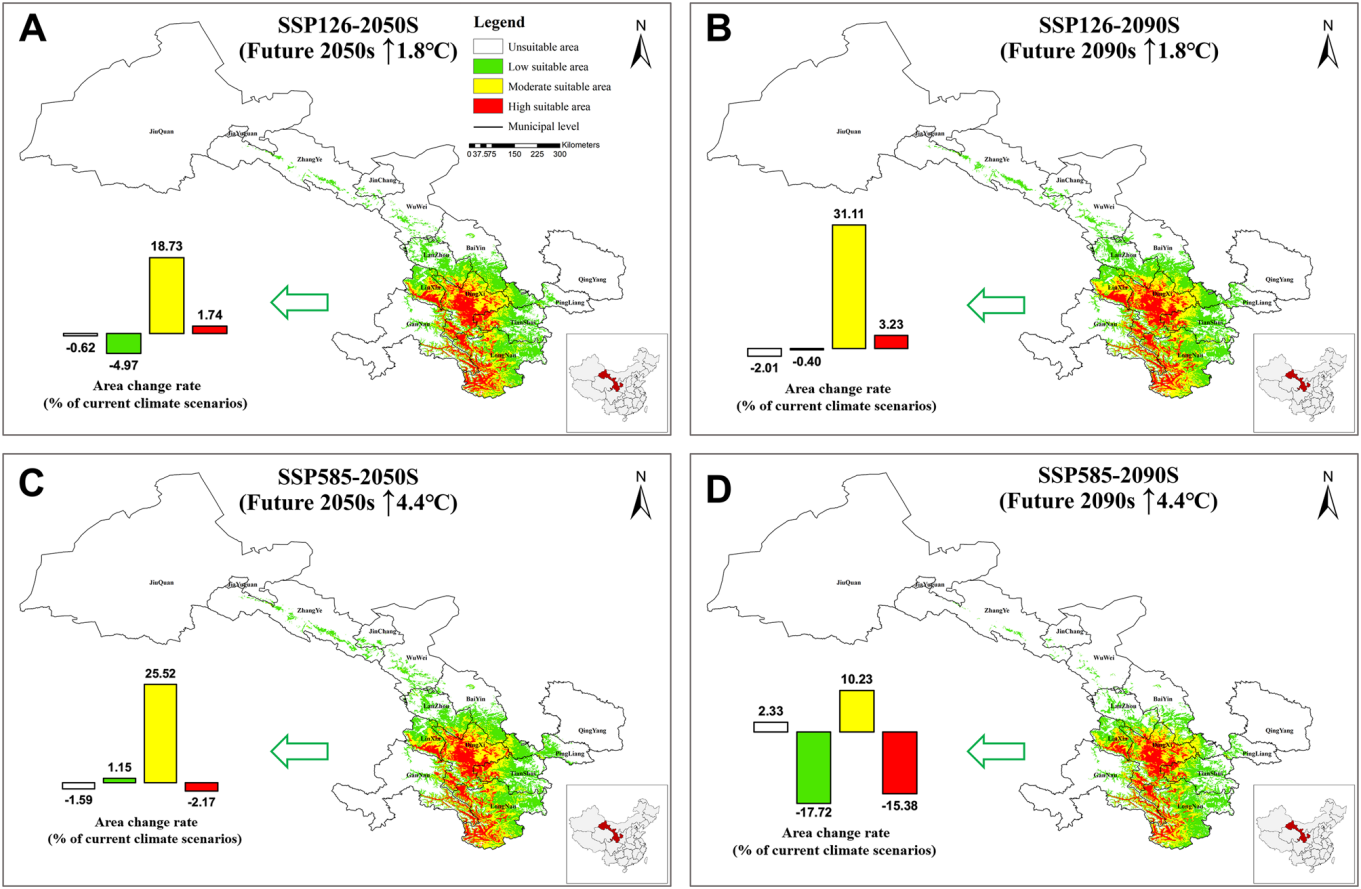
Suitable habitats for *Codonopsis pilosula* under different future climate scenarios (2050s and 2090s). (**A**) 2050 (average for 2041–2060), SSP126, (**B**) 2090 (average for 2081–2100), SSP126, (**C**) 2050 (average for 2041–2060), SSP585, (**D**) 2090 (average for 2081–2100), SSP585(The maps were prepared by Zixia Wang and Yanjun Jia in ArcGIS Pro, @@@).

Analyzed the changes in the suitable habitat of *Codonopsis pilosula* under climate change conditions (Fig. 2), calculated the change rate compared with the current suitable habitat of Codonopsis Radix (Fig. 2). Under the four future climate scenarios of SSP126-2050s, SSP585-2050s, SSP126-2090s, and SSP585-2090s, the moderately suitable areas showed an increasing trend, with an area of 2.52 × 10^4^ km^2^, 2.78 × 10^4^ km^2^, 2.66 × 10^4^ km^2^, and 2.34 × 10^4^ km^2^ respectively, with the change rates of 18.73%, 31.11%, 25.52%, and 10.23% respectively, and most of the increased moderate suitable areas were located in Dingxi City, Longnan City, and Linxia Hui Autonomous Prefecture. The highly suitable area showed an increasing trend under SSP126 climate scenario and a decreasing trend under SSP585 climate scenario, especially by 2090s, the area was reduced to 1.51 × 10^4^ km^2^, with a change rate of -15.38% (Fig. 2D). The increased highly suitable areas were mainly in Dingxi City and Tianshui City, while decreased in Longnan City and Linxia Hui Autonomous Prefecture. In addition, under the SSP585-2090s climate scenario, the low suitable area also showed a decreasing trend, and the area was reduced to 3.50 × 10^4^ km^2^, most of the reduced areas were located in Jinchang City, Wuwei City, Baiyin City, Pingliang City, and Qingyang City. In the different climate scenarios except for SSP585-2090s, the total suitable area of *Codonopsis pilosula* in Gansu province is increased.

### Analysis on quality zoning of Codonopsis Radix in Gansu Province based on all-in-one functional factor

**Figure 3:**
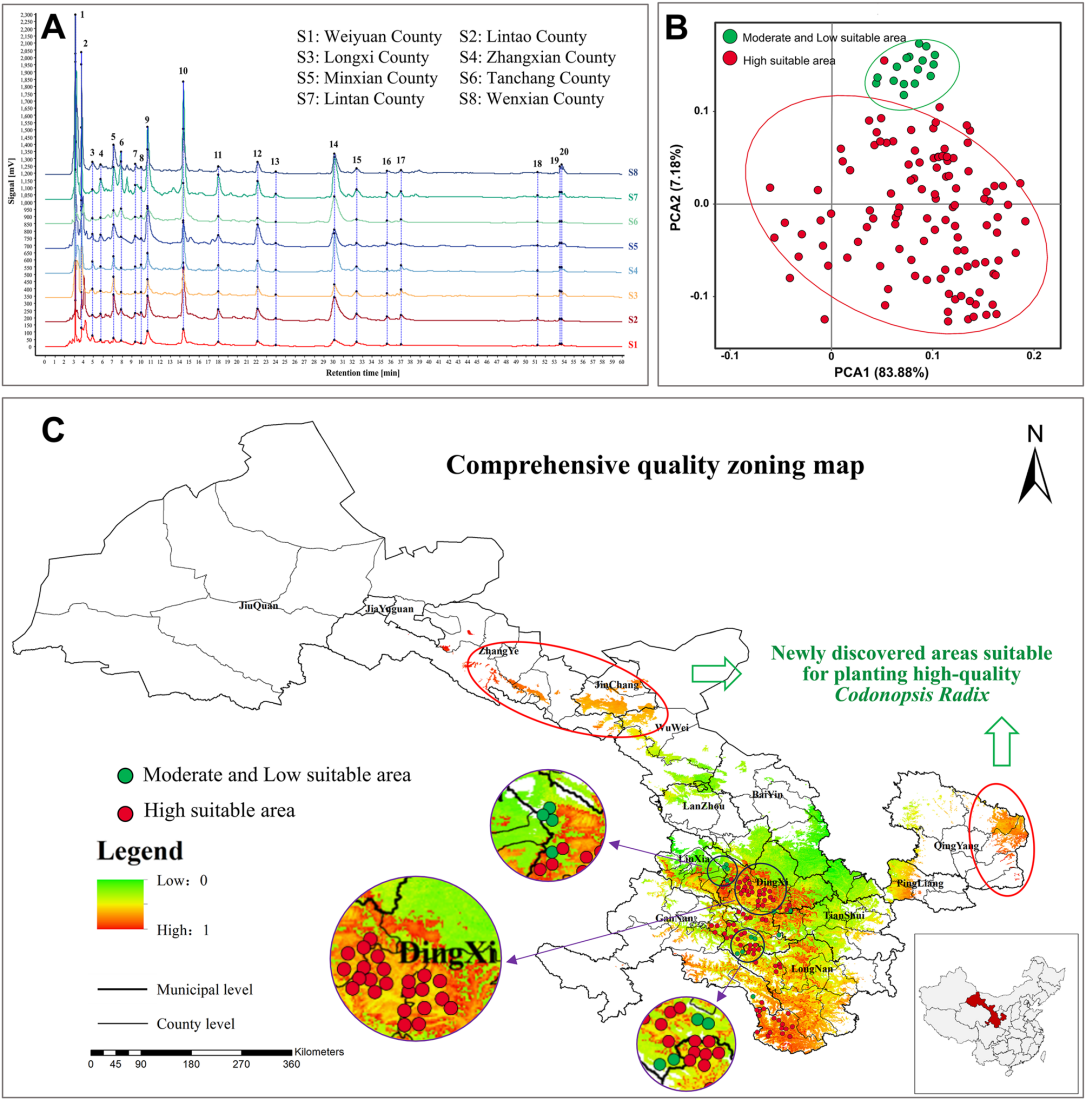
(**A**) The standard fingerprint of *Codonopsis Radix* in eight counties of Gansu Province, (**B**) PCA analysis of functional factors concentration between different suitable habitats, (**C**) The quality zoning map of *Codonopsis Radix* in Gansu Province(The maps were prepared by Zixia Wang and Yanjun Jia in ArcGIS Pro, @@@).

The chromatographic fingerprint of TCM can comprehensively reflect the types and quantities of chemical components contained in TCM and thus provide an overall description and evaluation of the quality of TCM. Sample S1 was selected for methodological validation, and the results showed that the relative standard deviation (RSD) of precision, repeatability, and stability were all less than 4.2%(required to be less than 5%), indicating that the established method is suitable for the establishment of the HPLC fingerprint of Codonopsis Radix. We imported the chromatograms of each sample into the "Similarity Evaluation System for Traditional Chinese Medicine Chromatographic Fingerprints, 2012 Edition"^30^ software and set the reference spectra and time window width. Through automatic matching and multi-point correction, we matched the common peaks and finally outputted the standard fingerprint. The standard chromatographic fingerprint of Codonopsis Radix from eight different districts in Gansu Province was shown in Fig. 3A (S1-S8 represent Weiyuan County, Lintao County, Longxi County, Zhangxian County, Minxian County, Tanchang County, Lintan County, and Wenxian County, respectively). Based on the matched 20 common peaks, a control map was generated and similarity was calculated. The results was shown in Table 3, indicating that the similarity values range from 0.649 to 0.975, indicating differences in the chemical composition content of Codonopsis Radix in different regions. The cumulative contribution rate of the five principal components obtained from principal component analysis reached 90.39%. This indicates that these 5 principal components have an overall interpretation rate of over 90%, which can represent the initial 20 common peak variables. The specific eigenvalues and contribution rates are shown in Table S4. Further calculating the principal component load matrix, the results are shown in Table S5. Common peaks with a contribution degree greater than 0.8 were selected for the five principal components, namely common peaks 1, 4, 5, 6, 9, 10, 11, 12, 14, 15, and 19. They will be used as indicators for subsequent correlation analysis.

**Table 3:** Similarity Values of *Codonopsis Radix* in eight districts of Gansu Province.

NO	S1	S2	S3	S4	S5	S6	S7	S8	R
S1	1.000								
S2	0.892	1.000							
S3	0.798	0.753	1.000						
S4	0.869	0.958	0.857	1.000					
S5	0.865	0.716	0.893	0.805	1.000				
S6	0.857	0.975	0.679	0.942	0.649	1.000			
S7	0.936	0.932	0.841	0.904	0.816	0.890	1.000		
S8	0.833	0.799	0.965	0.905	0.907	0.748	0.844	1.000	
R	0.943	0.945	0.905	0.970	0.889	0.907	0.966	0.933	1.000

Through the determination of the index components, effective compounds groups, nutritional components, nutrient elements, and the establishment of chromatographic fingerprint in 134 batches of Codonopsis Radix from different producing areas in Gansu Province, the results showed that there were some differences in the contents of lobetyolin, atractylenolide III, syringin, polysaccharides, oligosaccharides, alcohol extract, amino acids, protein, fat, dietary fiber, total nutrient elements and common peaks 1, 4, 5, 6, 9, 10, 11, 12, 14, 15, 19 in Codonopsis Radix from different producing areas. The habitat suitability values of 134 samples were extracted by ArcGIS and classified according to the classification rules under item 5.1(Table S6). Mann–Whitney U test indicated that the concentrations of the functional factors in Codonopsis Radix from different suitable areas are different (Fig. S3). The average concentrations of lobetyolin, polysaccharides, oligosaccharides, fat, common peak 1 area, common peak 5 area, common peak 10 area, common peak 12 area, and common peak 14 area from highly suitable areas were significantly higher than moderately suitable areas (*P* < 0.05). On the contrary, the average concentrations of alcohol extract and common peak 6 area from highly suitable areas were lower than in moderately suitable areas (*P* < 0.05). However, The average concentrations of atractylenolide III, syringin, amino acids, protein, dietary fiber, total nutrient elements, common peak 4 area, common peak 9 area, common peak 11 area, common peak 15 area, and common peak 19 area had no remarkable difference between the two categories (*P* > 0.05). Further, based on the all-in-one functional factor, the PCA method was used to study the geographical variation of Codonopsis Radix. As shown in Fig. 3B, the trend of separation according to the habitat suitability of the sample can be observed, indicating that there are differences in the chemical composition of Codonopsis Radix between different suitable habitats, which further illustrates the accuracy of the established MaxEnt model.

The correlation between functional factors and ecological factors was analyzed using SPSS. The concentrations of 22 functional factors were set as Y values, and the ten ecological factors screened were set as X values. Based on the results of stepwise regression analysis, the relationship model between functional factors and ecological factors was established (Table 4). The *P* value of the *F*-test result of the regression equation of each functional factor and the *T*-test result of the regression coefficient was significantly less than 0.05, indicating that the regression equation had a good prediction effect. The spatial analysis function of ArcGIS software was used to estimate the spatial distribution of functional factor concentration of Codonopsis Radix (Fig. S4). On this basis, based on the analysis results of habitat suitability, after removing the unsuitable distribution area, the concentration spatial distribution map of fingerprint common peaks, index components, and effective compounds groups was superimposed with the habitat suitability distribution map to obtain the medicinal quality zoning map of Codonopsis Radix in Gansu Province (Fig. S5). By superimposing the concentration spatial distribution map of nutritional components and nutritional elements with the distribution map of habitat suitability, the edible quality zoning map of Codonopsis Radix in Gansu Province was obtained (Fig. S6). Further, the quality zoning map of Codonopsis Radix in Gansu Province was obtained by superposing the 22 spatial distribution maps of the functional factors concentrations with the habitat suitability distribution map. Figure 3C illustrates visually that the comprehensive high-quality area of Codonopsis Radix was mainly distributed in Longnan and Longxi district of Gansu Province, such as Weiyuan County and Wenxian County. Two high-quality distribution areas suitable for *Codonopsis pilosula* planting were first found in the eastern part of Gansu Province while this area was not considered suitable for growing *Codonopsis pilosula* previously, such as Qingyang City and Pingliang City.

**Table 4:** Regression equation models between ecological factors and functional factors.

Functional factors	Regression equations
Lobetyolin	Y_1_ = 16.969–0.040X_1_-0.001X_2_-0.004X_3_ + 0.022X_4_ + 0.052X_5_-0.122X_6_ + 0.001X_7_ + 0.001X_8_-0.037X_9_ + 0.079X_10_
Atractylenolide III	Y_2_ = 52.840 + 1.287X_1_-0.007X_2_ + 2.748X_3_ + 1.785X_4_-0.460X_5_-1.695X_6_ + 0.143X_7_ + 0.013X_8_-2.351X_9_ + 0.269X_10_
Syringin	Y_3_ = 264.857–4.237X_1_-0.026X_2_ + 0.424X_3_ + 1.910X_4_ + 2.372X_5_-0.712X_6_ + 0.161X_7_ + 0.019X_8_-1.113X_9_-0.547X_10_
Polysaccharides	Y_4_ = 172.485 + 0.169X_1_-0.009X_2_-1.378X_3_-1.281X_4_-0.136X_5_-0.273X_6_ + 0.030X_7_-0.006X_8_-0.887X_9_ + 0.444X_10_
Oligosaccharides	Y_5_ = 25.193 + 0.148X_1_ + 0.001X_2_ + 0.684X_3_-0.648X_4_ + 0.436X_5_-1.116X_6_-0.033X_7_-0.001X_8_ + 0.045X_9_-0.297X_10_
Alcohol extract	Y_6_ = -89.105 + 0.459X_1_ + 0.013X_2_ + 0.579X_3_ + 0.489X_4_ + 0.417X_5_-1.017X_6_-0.015X_7_-0.003X_8_ + 0.507X_9_ + 1.623X_10_
Amino acid	Y_7_ = -157.827–1.546X_1_ + 0.006X_2_-0.239X_3_ + 3.612X_4_-2.817X_5_ + 6.634X_6_ + 0.121X_7_ + 0.029X_8_ + 2.206X_9_-3.624X_10_
Protein	Y_8_ = -53.384–0.065X_1_ + 0.003X_2_-0.130X_3_ + 0.633X_4_-0.373X_5_ + 0.984X_6_-0.030X_7_ + 0.003X_8_ + 0.532X_9_-0.348X_10_
Fat	Y_9_ = -2.803–0.013X_1_-0.012X_2_ + 0.064X_3_ + 0.085X_4_ + 0.015X_5_-0.024X_6_ + 0.001X_7_ + 0.001X_8_-0.001X_9_ + 0.002X_10_
Dietary fiber	Y_10_ = 61.543–0.145X_1_-0.007X_2_ + 0.495X_3_ + 1.445X_4_-1.141X_5_ + 1.778X_6_-0.013X_7_ + 0.010X_8_ + 0.724X_9_-0.259X_10_
Total nutrient elements	Y_11_ = 71.313 + 0.045X_1_-0.007X_2_-0.528X_3_ + 0.689X_4_ + 0.052X_5_-0.286X_6_ + 0.004X_7_ + 0.005X_8_-0.730X_9_ + 0.173X_10_
Peak 1 area	Y_12_ = -136.384 + 2.682X_1_ + 0.032X_2_-0.351X_3_-7.131X_4_-1.305X_5_ + 2.033X_6_-0.162X_7_-0.062X_8_ + 4.247X_9_-0.098X_10_
Peak 4 area	Y_13_ = -21.402 + 0.050X_1_ + 0.003X_2_ + 0.684X_3_-0.432X_4_ + 0.413X_5_-0.586X_6_-0.009X_7_-0.003X_8_-0.626X_9_-0.589X_10_
Peak 5 area	Y_14_ = 103.915 + 0.936X_1_ + 0.006X_2_ + 0.493X_3_-4.608X_4_-0.667X_5_-0.099X_6_ + 0.027X_7_-0.021X_8_ + 0.351X_9_-0.498X_10_
Peak 6 area	Y_15_ = 93.376–0.279X_1_-0.015X_2_-1.142X_3_ + 2.395X_4_-0.215X_5_ + 0.320X_6_-0.043X_7_ + 0.021X_8_ + 1.110X_9_-0.378X_10_
Peak 9 area	Y_16_ = 84.261 + 1.283X_1_ + 0.001X_2_-1.098X_3_-1.959X_4_-2.461X_5_ + 2.903X_6_ + 0.060X_7_-0.005X_8_ + 3.601X_9_-1.513X_10_
Peak 10 area	Y_17_ = 493.755–0.739X_1_-0.007X_2_-4.109X_3_-10.749X_4_-2.510X_5_ + 4.080X_6_ + 0.401X_7_-0.030X_8_ + 2.683X_9_ + 0.788X_10_
Peak 11 area	Y_18_ = 49.649–0.633X_1_ + 0.002X_2_ + 0.847X_3_-2.428X_4_ + 0.053X_5_ + 0.480X_6_ + 0.027X_7_-0.008X_8_ + 2.062X_9_-0.108X_10_
Peak 12 area	Y_19_ = 17.325–0.848X_1_ + 0.008X_2_ + 1.135X_3_-3.020X_4_-0.031X_5_ + 0.937X_6_-0.045X_7_-0.014X_8_ + 1.016X_9_ + 0.292X_10_
Peak 14 area	Y_20_ = 337.317–2.314X_1_ + 0.015X_2_ + 2.725X_3_-17.072X_4_ + 6.403X_5_-6.896X_6_ + 0.036X_7_-0.076X_8_-2.496X_9_ + 3.776X_10_
Peak 15 area	Y_21_ = 111.734–0.599X_1_-0.005X_2_-1.096X_3_-1.376X_4_ + 0.266X_5_ + 0.175X_6_ + 0.051X_7_-0.003X_8_ + 0.208X_9_ + 0.965X_10_
Peak 19 area	Y_22_ = 51.287–0.246X_1_-0.002X_2_-0.276X_3_-0.534X_4_-0.050X_5_ + 0.158X_6_ + 0.009X_7_-0.001X_8_ + 0.103X_9_ + 0.293X_10_

### Correlation analysis between ecological factors and functional factors of Codonopsis Radix

**Figure 4:**
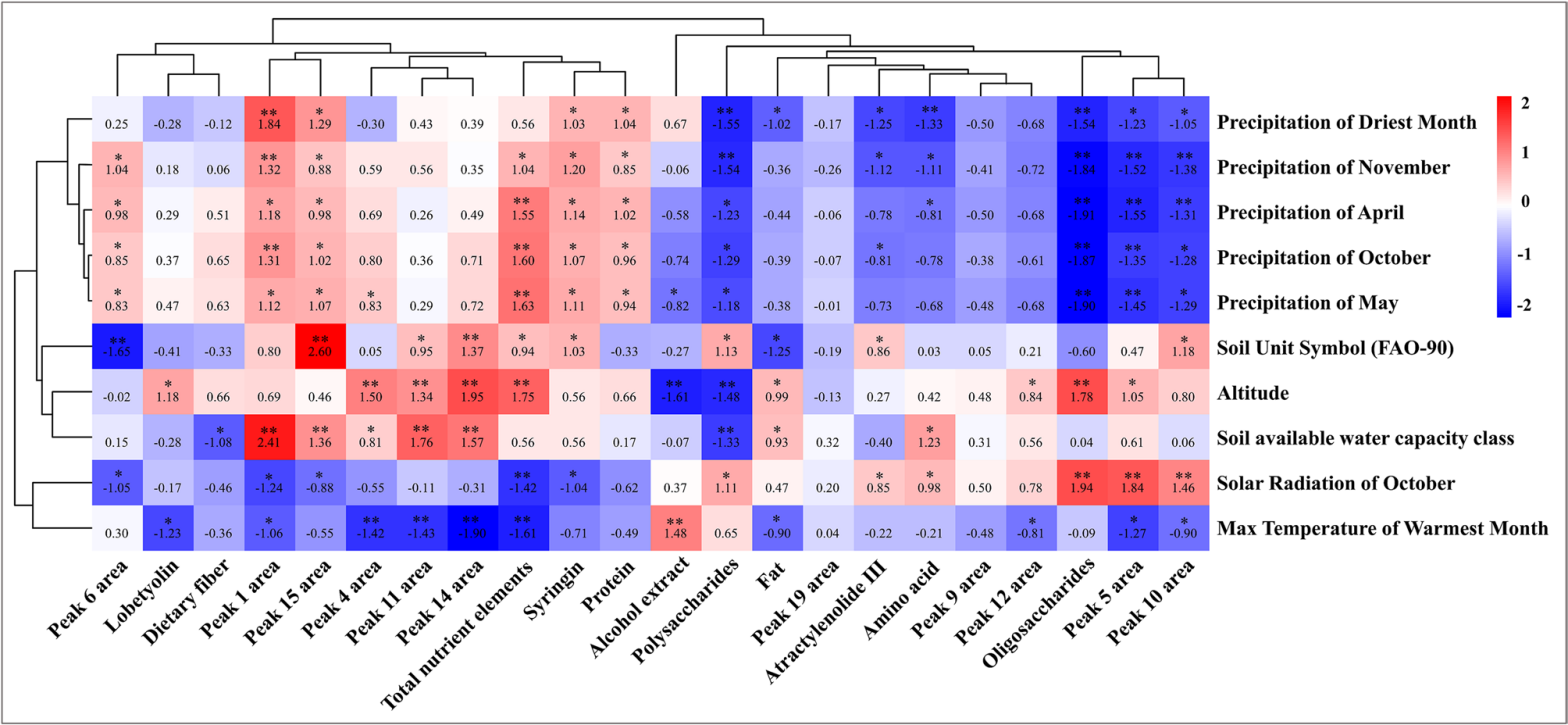
Correlation analysis between ecological factors and functional factors concentrations. Note: Significant differences (*: *P* < 0.05, **: *P* < 0.01).

Spearman correlation analysis results showed that the 10 main ecological factors had different influence on the accumulation of functional factors in Codonopsis Radix (Fig. 4). Firstly, the 10 ecological factors were divided into three categories by cluster analysis. Among them, the indicators related to precipitation were clustered into one category, including Precipitation of April, Precipitation of May, Precipitation of October, Precipitation of November, and Precipitation of Driest Month, the indicators related to temperature were clustered into one category, including Solar Radiation of October and Max Temperature of Warmest Month, the indicators related to soil topography were clustered into one category, including Soil Unit Symbol (FAO-90), Soil available water capacity class, and Altitude, indicating that the analysis results are reasonable and reliable. The functional factors of Codonopsis Radix, including lobetyolin, syringin, protein, dietary fiber, total nutrients, common peak 1 area, common peak 4 area, common peak 6 area, common peak 11 area, common peak 14 area, and common peak 15 area, were clustered into one group and was generally positively correlated with ecological factors such as precipitation. Atractylodes III, polysaccharides, oligosaccharide, alcohol extract, amino acid, fat, common peak 5 area, common peak 9 area, common peak 10 area, common peak 12 area, and common peak 11 area were clustered into one group, which was negatively related to precipitation ecological factors. In addition, there was a highly significant relationship between the common peak 6 area, common peak 15 area, and common peak 14 area with Soil Unit Symbol (FAO-90) (*P* < 0.01). The increase of altitude was beneficial to the increase of oligosaccharide, total nutrient elements, common peak 4 area, common peak 11 area, and common peak 14 area (*P* < 0.01), but was not conducive to the accumulation of polysaccharide and alcohol extracts of Codonopsis Radix*.*

## Discussion

This study combined MaxEnt model and GIS technology to analyze the habitat suitability distribution of *Codonopsis pilosula* in Gansu Province. The predicted AUC value was high, and the predicted potential suitable distribution area was basically consistent with the distribution of *Codonopsis pilosula* recorded in the literature^11^, indicating that the potential distribution of species predicted by MaxEnt model had high accuracy. When we established MaxEnt model, we found that the maximum temperature, minimum temperature, water vapor pressure, wind speed, and solar radiation from January to December were not used as ecological factors in the analysis of habitat suitability distribution of *Codonopsis pilosula*^26^. The research showed that the maximum temperature, minimum temperature^42,43^, water vapor pressure, wind speed^44^, and solar radiation^45^ were important factors affecting the growth and distribution of species. The various ecological factors have important impact on distribution of species, we imported 115 ecological factors into the MaxEnt model for analysis including 19 bioclimatic variables, temperature, precipitation, soil, topography, etc. After the screening, 10 factors were identified as the main ecological factors affecting the distribution of *Codonopsis pilosula*. *Codonopsis pilosula* is a deep-rooted plant, suitable for growing in deep, loose, well-drained soil^46^. *Codonopsis pilosula* is usually sown from the middle of April to the early of May, transplante after the soil is completely thawed from the middle of March to the middle of April in early spring or from the middle of September to the end of October in autumn and harvest from the late of October to the early November^47^. The precipitation in April and May plays a vital role in the germination of seeds and the growth of seedlings after sowing and the growth of *Codonopsis pilosula* seedlings during transplanting. Insufficient precipitation will lead to reduced germination rate and low-quality *Codonopsis pilosula* seedlings^48^. October precipitation and solar radiation are also essential ecological conditions for *Codonopsis pilosula* transplanting and a vigorous growth period. Furthermore, some studies have found that *Codonopsis pilosula* can grow normally at a temperature of 8 ~ 30 °C, suitable for growth at 15 ~ 25 °C, vigorous growth at about 19 °C, and when temperature is above 30 °C the growth was inhibited^48^. In this study the max temperature of warmest month suit for *Codonopsis pilosula* growing is 20.2 ~ 29.3 °C, which was consistent with above literature. The water resources in Gansu Province include the Yellow River Basin, Yangtze River Basin, and inland river basin. The suitability areas of *Codonopsis pilosula* obtained in this study were mainly distributed in the Weihe River System in the Yellow River Basin and the Bailong River System in the Yangtze River Basin, while the unsuitable areas were mainly high-altitude mountainous areas, deserts, and Gobi areas. Weihe River System^49^ and Bailong River System^50^ flowing through the real estate area are the main producing areas of *C. pilosula* and *C. pilosula* var. *modesta* in Gansu Province. The study found that the suitable area of the Bailong River system was located at an altitude of 568 ~ 3049 m, and the slope was suitable, while the unsuitable area was the high mountain with high altitude and poor soil quality, which was not conducive to the growth of *Codonopsis pilosula*. In addition, Zhangye City, Jinchang city, and Wuwei City, which have been proved to be unsuitable for the growth of *Codonopsis pilosula* by previous studies^51^. After comprehensive analysis of various types of ecological factors, we found that some areas in Zhangye City, Jinchang city and Wuwei city were suitable for the growth, indicating that the climatic conditions satisfied the needs of the normal growth and development of *Codonopsis pilosula*, and an appropriate scale of *Codonopsis pilosula* can be planned and planted in these areas.

In the analysis of the potential suitability distribution of *Codonopsis pilosula* under climate change conditions, we found that the highly suitable area showed an increasing trend under the SSP126 climate scenario (when the global temperature rises by 1.8 °C), and a decreasing trend under the SSP585 (when the global temperature rises by 4.4 °C) climate scenario. This was consistent with the implication of SSP126 (described as the sharp reduction of global carbon dioxide, the transformation of the economy to sustainable development) and SSP585 (described as the rapid economic growth driven by the exploitation of fossil fuels and the implementation of energy-intensive lifestyle)^52^ models. The highly suitable area of *Codonopsis pilosula* located in the Bailong River system will be reduced, and the highly suitable area of the Weihe River system will be transferred to Tongwei county and Anding District in the northeast of Dingxi City under climate change conditions. The possible reason is that with the increasing demand for Codonopsis Radix, the demand for cultivated land is also increasing. People prefer to introduce and cultivate *Codonopsis pilosula* in low-altitude producing areas with convenient planting conditions, such as most *Codonopsis pilosula* producing areas and some new producing areas in Dingxi, Gansu Province, which brings considerable pressure to the sustainable survival of *Codonopsis pilosula* in high-altitude areas. At the same time, due to the longer growth cycle of *Codonopsis pilosula* than other crops, some high-altitude *Codonopsis pilosula* production areas have been replaced by other crops, which has also been proved by our field surveys, so species at high altitudes are more likely to be affected by climate change.

In this study, the quality of Codonopsis Radix from different producing areas was comprehensively evaluated by measuring the index components, functional component groups, nutrient components, and nutrient element contents and establishing fingerprints. The results showed that there were differences in the functional factors of Codonopsis Radix in different producing areas and different suitable areas of *Codonopsis pilosula* had a significant impact on the accumulation of functional factors, indicating that the highly suitable area as the planting area of *Codonopsis pilosula* can provide high-quality TCM^53^. Considering that the ecological environment is the result of the comprehensive action of various ecological factors, a regression model between ecological factors and functional factors was established to evaluate the changes of Codonopsis Radix quality in different ecological environments as a whole. The results showed that no matter the comprehensive quality zoning map of Codonopsis Radix or the medicinal and edible quality zoning map of Codonopsis Radix, most of the high-quality areas were located in the high suitability area of *Codonopsis pilosula*, while the low-quality areas were located in the low suitability area, indicating that the suitability of the ecological suitability area was related to the spatial quality changes of functional factors of Codonopsis Radix, and the suitable growth area was also conducive to the production and accumulation of secondary metabolites of *Codonopsis pilosula*. For example, Wenxian County, Wudu District, and surrounding counties in Gansu Province are the main producing areas of *C. pilosula* var. *modesta*, Lintao County, Weiyuan County, Longxi County, Zhangxian County and surrounding counties in Gansu Province are the main producing areas of *C. pilosula*. It is not only a highly suitable growth area for *Codonopsis pilosula* but also a comprehensive high-quality area for Codonopsis Radix. It can be seen that the quality of *C. pilosula* var. *modesta* produced in Wenxian County and surrounding counties and districts is higher than that of *C. pilosula* produced in other producing areas of Gansu Province, which is consistent with the popularity of people caused by the efficacy and yield of Codonopsis Radix in different producing areas and the long-term price formed in the circulation of Codonopsis Radix market. The growth conditions of *C. pilosula* var. *modesta* are more demanding, requiring planting land above 2000 m above sea level, and then growing for 4~6 years and then digging, so it is a semi-wild state. Its nutritional value is higher than that of ordinary *Codonopsis pilosula* and has better spleen and stomach effects. In addition, the smell of *C. pilosula* var. *modesta* will be stronger, and its effect of tonifying qi and blood will be stronger^54^. Based on the comprehensive efficacy value, nutritional value, planting conditions, and other factors, the price of *C. pilosula* var. *modesta* is higher than that of ordinary *Codonopsis pilosula*. Our previous field investigation also found that the current price of *C. pilosula* var. *modesta* is RMB 300~500 per kilogram, while the price of *C. pilosul* with a wide distribution of origin is RMB 100~200.

We found that Codonopsis Radix in some low suitable areas showed higher quality, such as Kang County and Cheng County in Longnan City, and some areas of Qingyang City, Zhangye City, and Jinchang City, which may be related to environmental stress on plants. The ecological environment can be divided into prosperity and adversity. It is generally believed that prosperity is conducive to the growth of species and the accumulation of chemical components. However, in recent years, more and more studies have shown that species under the influence of environmental stress will produce some secondary metabolites to strengthen their adaptability to adversity and promote the formation of genuine medicinal materials^55^, for example, under the stress of high temperature, high humidity, and low-dose potassium deficiency, the proportion of naphtha components of *Atractylodes lancea* is closer to the proportion of naphtha components in genuine areas^56^. The correlation analysis between different environmental factors and functional factors showed that precipitation, temperature and altitude had the greatest influence on the quality of Codonopsis Radix. *Codonopsis pilosula* in Gansu Province is mostly cultivated and less wild. Based on some areas of *Codonopsis pilosula* under environmental stress in Gansu Province, making full use of its unique ecological and environmental conditions, we can explore reasonable planting methods for high-yield and high-quality *Codonopsis pilosula*.

## Conclusions

**Figure 5:**
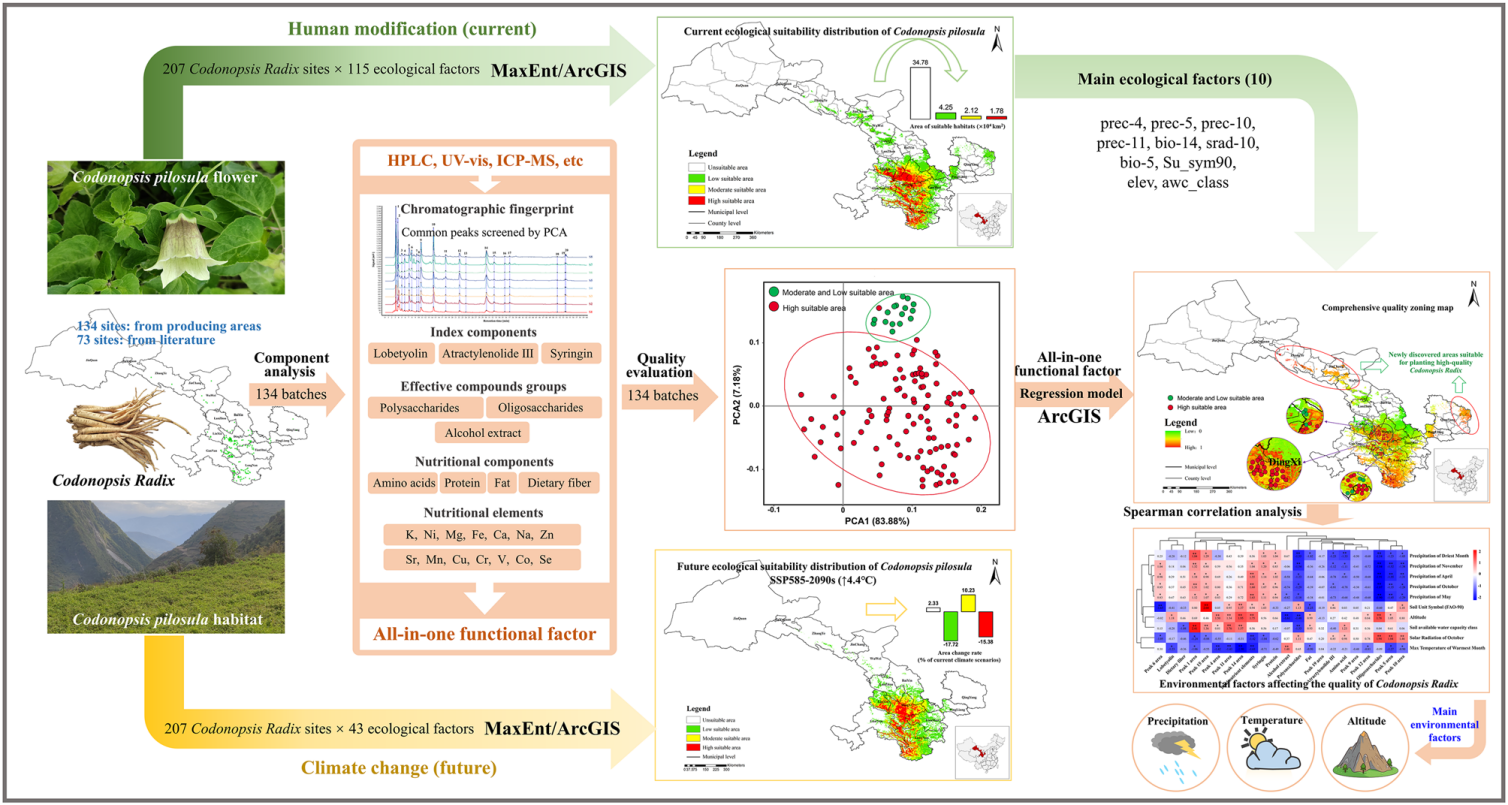
Research process and results of the article.

In this study, a high-precision MaxEnt model was successfully established. The suitable ecological area for the growth of *Codonopsis pilosula* was clarified. The quality of Codonopsis Radix was systematically analyzed based on fingerprint, functional components, nutrients, and nutrient elements, and the quality and ecological environment model of *Codonopsis pilosula* was established based on the all-in-one functional factor, and the quality zoning of Codonopsis Radix was constructed. The comprehensive high-quality area of Codonopsis Radix has a certain correlation with the highly suitable area of *Codonopsis pilosula*. Temperature, precipitation, and altitude are the most important environmental factors affecting the quality of Codonopsis Radix*.* The spatial distribution and changes of the suitable area of *Codonopsis pilosula* under the future climatic conditions were further evaluated. By 2090s, the area of the highly suitable area of *Codonopsis pilosula* will be significantly reduced compared with the current situation. The overall research results are shown in Fig. 5.This study provides a new perspective and strategy for evaluating the influence of climate factors on the quality of Codonopsis Radix and optimizing the planting layout of *Codonopsis pilosula*.

## Materials and methods

### Habitat suitability analysis of *Codonopsis pilosula*

#### Codonopsis Radix distribution points data

The Codonopsis Radix distribution points data were obtained in two channels: (1) field collection. Collect Codonopsis Radix samples on the spot and record the GPS information of the place where the samples are collected, including longitude, latitude, (2) historical data. Through Global and Chinese online databases, such as Chinese Virtual Herbarium (CVH, http://www.cvh.ac.cn/), Global Biodiversity Information Facility (GBIF, https://www.gbif.org/), to obtain the historical growth information of Codonopsis Radix. About 245 occurrence data points for the Codonopsis Radix were collected in this study, involving 11 cities and 24 counties in Gansu Province. In order to avoid overfitting the model, the data was imported into ArcGIS 10.2 software, only one occurrence data point was retained in the 1 km environmental grid data, and the suspected wrong occurrence points were deleted. Finally, 207 occurrence data were screened, of which 134 were from field collection, and 73 were from historical data. The occurrence data is sorted in the excel table according to the three columns of species name, longitude, and latitude, and stored as a.csv format file to meet the requirements of the MaxEnt software.

#### Environmental variables

The ecological factors used in this study were shown in Table 1. The 103 climate-type data were from the WorldClim-Global Climate Data (https://www.worldclim.org/) (1970–2000). Future climate data were obtained from one global climate model (BCC-CSM2-MR, the Beijing Climate Center Climate System Model), and SSP126 and SSP585 in two simulation cycles 2041–2060 (2050s) and 2081–2100 (2090s) were selected, the ecological factors involved were bioclimatic variables (bio1-19), and monthly precipitation from January to December (prec1-12). Data of 8 soil types, from the Harmonized World Soil Database (HWSD, https://www.fao.org/home/en/), the format was grid data with a spatial resolution of 1 km, including soil types, soil physical, and chemical properties, etc. Topographic data were obtained from Geospatial Data Cloud elevation data (DEM) (http://www.gscloud.cn/) (the spatial resolution is 30 m), and the slope and aspect were generated by the surface analysis function of ArcGIS. Vegetation type data is provided by “Environmental & Ecological Science Data Center for West China, National Natural Science Foundation of China” (http://westdc.westgis.ac.cn/), which was made from the vegetation subclass data. Considering that the soil, terrain, and vegetation will not change much under climate change conditions, the corresponding data obtained at present were still applicable to the future. A layer format of ASCII by ArcGIS software (version 10.2), so that it can be loaded into MaxEnt.

#### Species distribution modeling process

The prediction model of *Codonopsis pilosula* habitat suitability distribution area was established with maximum entropy model software (MaxEnt 3.4.4). Import 115 ecological factor data and 207 Codonopsis Radix distribution point data into MaxEnt, set 25% of the distribution point data as the testing dataset, and 70% were the training dataset, the maximum number of iterations was 10^4^, and set the analysis omission/debugging curve, receiver operating curve (ROC), jackknife method and response curve^57^. This research used the area under the ROC curve (AUC) and the analysis omission/debugging curve to verify the accuracy of the model prediction results. Where the omission rate provides information on the model under and over-fitting, the test omission rate should be consistent with the theoretical omission rate for a good model^58^. When the AUC value was less than 0.6, the model prediction fails, poor (less than 0.7), good (between 0.7 and 0.9), and more than 0.9, the model prediction was excellent and the accuracy was high^59^.

To find out which variables were most important to the *Codonopsis pilosula* being modeled, we imported the distribution point data of Codonopsis Radix and the data of 115 ecological factors into MaxEnt for iterative calculation, and discarded the ecological factors with a contribution rate of 0 in the calculation results, and continued to iterate until all ecological factors contributed. The value of ecological factors with contribution was analyzed by Pearson correlation analysis through SPSS 22.0 to obtain the correlation coefficient. When the correlation coefficient was greater than 0.8, the smaller contribution rate of ecological factors was discarded, analyzed the remaining ecological factors by MaxEnt. Combined with the percent contribution, permutation importance, and the weight of each ecological factor in Jackknife test, the common ecological factor was taken as the main ecological factor and ran the model 10 times. we took the average value to analyze the habitat suitability of *Codonopsis pilosula* and obtained the suitability range of main ecological factors through the response curve. This research used the natural breaks method (Jenks)^60^ to divide the suitable areas of *Codonopsis pilosula* distribution: unsuitable area (0, 0.1), low suitable area (0.1, 0.3), moderate suitable area (0.3, 0.6), and high suitable area (0.6, 1.0). Meanwhile, drew the habitat suitability distribution map of *Codonopsis pilosula* in Gansu Province, and calculated the area of each suitable area by using the regional analysis function in ArcGIS.

#### Habitat suitability analysis of *Codonopsis pilosula* under climate change conditions

Based on the data of climatic factors under four future climate scenarios SSP126-2050s, SSP585-2050s, SSP126-2090s, and SSP585-2090s, and other ecological factors screened, the potential habitat suitability of Codonopsis Radix under climate change conditions was analyzed.

### Quality zoning analysis based on the all-in-one functional factor of Codonopsis Radix

#### Instruments and reagents

Instruments: High performance liquid chromatograph (Agilent 1260 II), equipped with a DAD detector; Ultrasonic cleaning instrument (Ningbo Xinzhi Biotechnology Co., Ltd.); Rotary evaporator (Zhengzhou Greatwall Science Industry and Trade Co., Ltd.); 1/100,000 Analytical balance (Sartorius Scientific Instruments Beijing Co., Ltd.); Constant temperature electric heating plate (Shanghai Keheng Industrial Development Co., Ltd.); Constant temperature water bath box (Shanghai Yiheng Scientific Instrument Co., Ltd.); Circulating water multi-purpose vacuum pump (Zhengzhou Greatwall science industry and Trade Co., Ltd.); Centrifuge (Hitachi, Japan); Freeze dryer (labconco, USA); Uv-1700 Ultraviolet–visible spectrophotometer (Shimadzu, Japan); Agilent 7900 inductively coupled plasma mass spectrometer (ICP-MS, Agilent Technologies, Santa Clara, USA); S-433D automatic amino acid analyzer (SYKAM); N-WYVAP 112 Termovap Sample Concentrator (Organomation); DigiPREP TKN Systems Kjeltec 8400 (FOSS, Denmark).

Reagents: Three standards (lobetyolin, atractylenolide III, syringin) were purchased from Sigma-Aldrich. Chromatographic grade methanol and acetonitrile were purchased from Guangzhou Aixin Scientific Instrument Co., Ltd. (Guangzhou, China), other chemical reagents are analytical grade, and ultra-pure water is re-distilled self-made deionized water. Standard (D-glucose) was purchased from the National Institutes for Food and Drug Control. Amino acid reference substances (Ala, Arg, Asp, Met, Cys, Glu, Gly, Lys, His, le, Leu, Tyr, Phe, Pro, Ser, Thr, Val) were purchased from SIGMA-ALDRICH. Catalyst sheet (FOSS, Denmark), Thermal stability α- amylase (ShanghaiyuanyeBio-TechnologyCo., Ltd.), Alkaline protease (Beijing SuoLaibao Technology Co., Ltd.), Starch glucosidase (Shanghai Macklin Biochemical Co., Ltd.), Multi-element standard solution (Inorganic Ventures, USA).

Codonopsis Radix sample: In this study, 134 batches of Codonopsis Radix samples were collected from Weiyuan County, Lintao County, Longxi County, Zhangxian County, Minxian County, Tanchang County, Lintan County and Wenxian County in Gansu Province. The samples were identified by Professor Hu Fangdi of Lanzhou University as the roots of *Codonopsis pilosula* (Franch.) Nannf. (*C. pilosula*) and *Codonopsis pilosula* var. *modesta* (Nannf.) L.T.Shen (*C. pilosula* var. *modesta*). Specific information is shown in Table S2. After cleaning and drying, the Codonopsis Radix samples were crushed into powder and stored in a dry self-sealing bag for various functional factor analysis.

#### Establishment of fingerprint

The extraction adopts the method specified in the pharmacopeia (2020 version). The purification method was performed by a self-built laboratory method: dissolved the evaporated extract in 5 mL of 45% ethanol, and use a macroporous adsorption resin column (specification: 20 mm × 400 mm) saturated for 5 min, first eluted with 200 mL of distilled water (elution rate of 0.5 mL/min), discarded the eluent, and then eluted with 150 mL of anhydrous ethanol (elution rate of 0.5 mL/min). Collected the ethanol eluent, evaporated to dryness, dissolved in methanol, and diluted to 1 mL. Added 10 μL of the sample solution into a high-performance liquid chromatograph and determined its fingerprint. Conducted precision experiments, repeatability experiments, and stability experiments on sample S1, and conducted methodological investigations on chromatographic conditions based on the main characteristic peak area.

Chromatographic conditions: Chromatographic separation of the sample solution was achieved on a Diamonsil C18 column, and the organic phase (A) was 100% acetonitrile, (B) was 0.2% acetic acid aqueous solution, and the flow rate was 1.0 mL/min, the column temperature (30 °C), and the detection wavelength was set at 267 nm and 320 nm, the injection volume (20 µL). Gradient elution procedure: 10 → 20% A (from 0 min to 15.00 min), 20%A (from 15.00 min to 25.00 min), 20 → 30% A (from 25.00 min to 35.00 min), 30 → 45% A (from 35.00 min to 45.00 min), 45 → 90% A (from 45.00 min to 50.00 min).

We selected representative chromatograms of Codonopsis Radix samples from 8 districts, imported them into the "Similarity Evaluation System for Traditional Chinese Medicine Chromatographic Fingerprints 2012 Edition", processed and analyzed the chromatograms, generated common pattern chromatograms of Codonopsis Radix from different districts, and used the common pattern chromatograms as the control chromatograms. Through multi-point correction and automatic matching, common peaks were determined, and similarity evaluation was conducted on Codonopsis Radix from 8 districts^61^. This study used SPSS software for principal component analysis. Firstly, the common peak areas of the samples were imported into SPSS for data standardization processing; Then perform factor analysis to calculate the corresponding eigenvalues and contribution rates of the principal components, and screen the principal components based on the principle of eigenvalues ≥ 1^62^; Finally, common peaks with significant contributions to the principal component were selected based on the load factor and principal component score coefficient for subsequent correlation analysis^63^.

#### Index component

Sample preparation was performed in the light of a previously reported method, and made appropriate adjustments. The preparation method for the analysis samples of lobetyolin and atractylenolide III is the same: sample powder (The powder of Codonopsis Radix (2.0 g) was added with 20 mL methanol for reflux extraction. After filtration, 16 mL methanol was added for reflux extraction, 40 min each time. The filtrate was combined twice and concentrated to 10 mL. Finally, the filtrate was treated with 0.45 μm filter membrane and took 1.5 ml for analysis. The analytical samples of syringin: dried sample powder was accurately weighted (1.0 g) and extracted with 25 mL 75% methanol–water solution for 45 min by an ultrasound-assisted method. The filtrate was treated with 0.45 μm filter membrane and took 1.5 ml for analysis. Preparation of reference substance: Accurately weighed 1.13 mg, 1.04 mg, and 0.22 mg of the reference substances of lobetyolin, atractylenolide III, and syringin respectively, and dissolved into 0.565 mg/ml,0.52 mg/ml, and 0.11 mg/ml solution with methanol. The following methods are self built in the laboratory. The chromatographic conditions used were as follows. Lobetyolin and Atractylenolide III: Chromatographic separation of the sample solution was achieved on a Diamonsil C18 column (250 × 4.6 mm, 5 μm), and the organic phase was acetonitrile–water (Lobetyolin, 26:74, Atractylenolide III, 65:35), the column temperature (30 °C), the injection volume (10 µL), the flow rate (1.0 mL/min), and the detection wavelength was set at 267 nm (Lobetyolin), 220 nm (Atractylenolide III)^64^. Syringin: Chromatographic separation of the sample solution was achieved on a Kromasil C18 column (250 × 4.6 mm, 5 μm). The organic phase (A) was 100% acetonitrile, (B) was 0.1% phosphoric acid solution, and the flow rate was 0.8 mL/min. Gradient elution procedure: 10% A (from 0.00 to 20.00 min), 10 → 30% A (from 20.00 to 30.00 min), 30 → 70%A (from 30.00 to 50.00 min). The column temperature, the injection volume, and the detection wavelength were set the same as Atractylenolide III^65^. Injected each reference solution and sample solution into the HPLC, drew the standard curve from the measured concentration of the reference, and determined the concentration of lobetyolin, atractylenolide III, and syringin according to the standard curve.

#### Effective compounds groups

The polysaccharides and oligosaccharides were extracted by water extraction and alcohol precipitation. Accurately weighted 10.0 g Codonopsis Radix powder, and extracted with tenfold of 95% ethanol for two times, and each time for 1 h, discarded the filtrate. The dried residues after ethanol extraction were further extracted twice with tenfold volume of distilled water, for 45 min each time. The extracts were combined and concentrated to 1/4 of the original volume. According to the volume of the concentrated solution, an appropriate amount of 95% ethanol was slowly added to the concentrated solution, so that the final concentration of ethanol in the solution was 80%, and placed overnight. Centrifuged for 15 min with 10,600 × g centrifugal force, collected the precipitate, freeze-dried to constant weight, and got the polysaccharides. Simultaneously, collected the supernatant after centrifugation, concentrated and recovered ethanol under reduced pressure at 50 °C, 60r/min. The residue was freeze-dried to constant weight and got the oligosaccharides^66^. Then, determined the concentration of polysaccharides and oligosaccharides by the phenol–sulfuric acid assay^67^. Alcohol extract concentration determination was based on the procedures of the Chinese Pharmacopeia (2020 version)^68^.

#### Nutrients and elements

Amino acids concentration was determined by S-433D automatic amino acid analyzer^69^, the protein concentration in Codonopsis Radix was detected by Kjeldahl technique^70^, determined the fat concentration by acid hydrolysis method^71^, the concentration of dietary fiber was determined by enzymatic–gravimetric method^72^. Agilent 7900 inductively coupled plasma mass spectrometer (ICP-MS) (Agilent Technologies, Santa Clara, USA) was used to determine 14 nutrient elements (K, Ni, Mg, Fe, Ca, Na, Zn, Sr, Mn, Cu, Cr, V, Co, Se)^73^.

#### Quality zoning analysis of Codonopsis Radix

For the sake of studying the effect of habitat suitability on functional factors accumulation, we extracted the ecological factor value of each occurrence point through ArcGIS according to the longitude and latitude information of Codonopsis Radix, and the common peaks area of fingerprint spectrum, the average concentration of index components, effective compounds groups, nutritional components, and nutritional elements were compared to highly suitable areas with moderately suitable areas. Mann–Whitney *U* test was used to evaluate the concentration change of functional factors between different suitable regions. In order to explore the overall variability of Codonopsis Radix in different habitats, we constructed the all-in-one functional factor based on 22 functional factors in Codonopsis Radix, and used the principal component analysis (PCA) model to predict the origin of samples (highly suitable area and moderately suitable area) by using the all-in-one functional factor of 134 batches of Codonopsis Radix after processing. The ecological environment is the result of the comprehensive action of various ecological factors. Therefore, using the stepwise regression analysis method of SPSS software, the above-screened ecological factors were taken as a whole to measure the impact of ecological factors on the functional factors of Codonopsis Radix, and the regression model was established. Based on the model of each functional factor, the spatial distribution of the concentration of each functional factor was analyzed by ArcGIS. And the obtained functional factors spatial distribution and suitability maps were processed uniformly, and a linear function was selected to obtain a numerical range of 0–1 for all layers. Then, fuzzy superposition analysis was used to obtain the quality zoning map of Codonopsis Radix in Gansu Province. At the same time, from the medicinal and edible aspects of Codonopsis Radix, the common peaks area of the fingerprint spectrum, the index components, and effective compounds groups were used as the indicators to evaluate the quality of Codonopsis Radix when it was used as medicine, and the nutritional components and nutritional elements were used as the indicators to evaluate the quality of Codonopsis Radix when it was used as food. Finally, it was superimposed with the habitat suitability distribution map of Codonopsis Radix and obtained the medicinal quality zoning map of Codonopsis Radix and the edible quality zoning map of Codonopsis Radix in Gansu Province. Further, we used Spearman correlation analysis to study the influence of ecological factors on the concentration of functional factors of Codonopsis Radix based on a single ecological factor variable.

### Supplementary Information

Supplementary Figures.

Supplementary Tables.

## Data Availability

The authors confirm that the data supporting the findings of this study are available within the article [and/or its supplementary materials].
