# Supplementary material for: Study on environmental factors affecting the quality of codonopsis radix based on MaxEnt model and all-in-one functional factor
Source: Sci Rep. 2023 Nov 25;13:20726. doi: 10.1038/s41598-023-46546-6 (PMC10676394; doi:10.1038/s41598-023-46546-6)
Supplement: Supplementary file 1 — Supplementary Figures. [file 41598_2023_46546_MOESM1_ESM.docx]

**Study on environmental factors affecting the quality of *Codonopsis Radix* based on MaxEnt model and** **all-in-one** **functional factor**

**Zixia Wang^a#^，Yanjun Jia^a#^，Pengpeng Li^a^，Zhuoshi Tang^a^，Yina Guo^a^，Longxia Wen^a^，Huaqiao Yu^a^，Fang Cui^a,b,c,d^，Fangdi Hu^a,b,c,d*^**

^a^School of Pharmacy, Lanzhou University, Lanzhou, 730000, China

^b^State Key Laboratory of Applied Organic Chemistry, Lanzhou University, Lanzhou, 730000, China

^c^Codonopsis Radix Research Institute, Gansu Province, Lanzhou, 730000, China

^d^Codonopsis Radix Industrial Technology Engineering Research Center, Gansu Province, Lanzhou, 730000, China

^#^Yanjun Jia and Zixia Wang contributed equally to this work

^*^ Corresponding author.

E-mail: hufd@lzu.edu.cn; Fax: +860931 8915686; Tel: +860931 8911865/8911895

**Supplementary material (figure)**

**
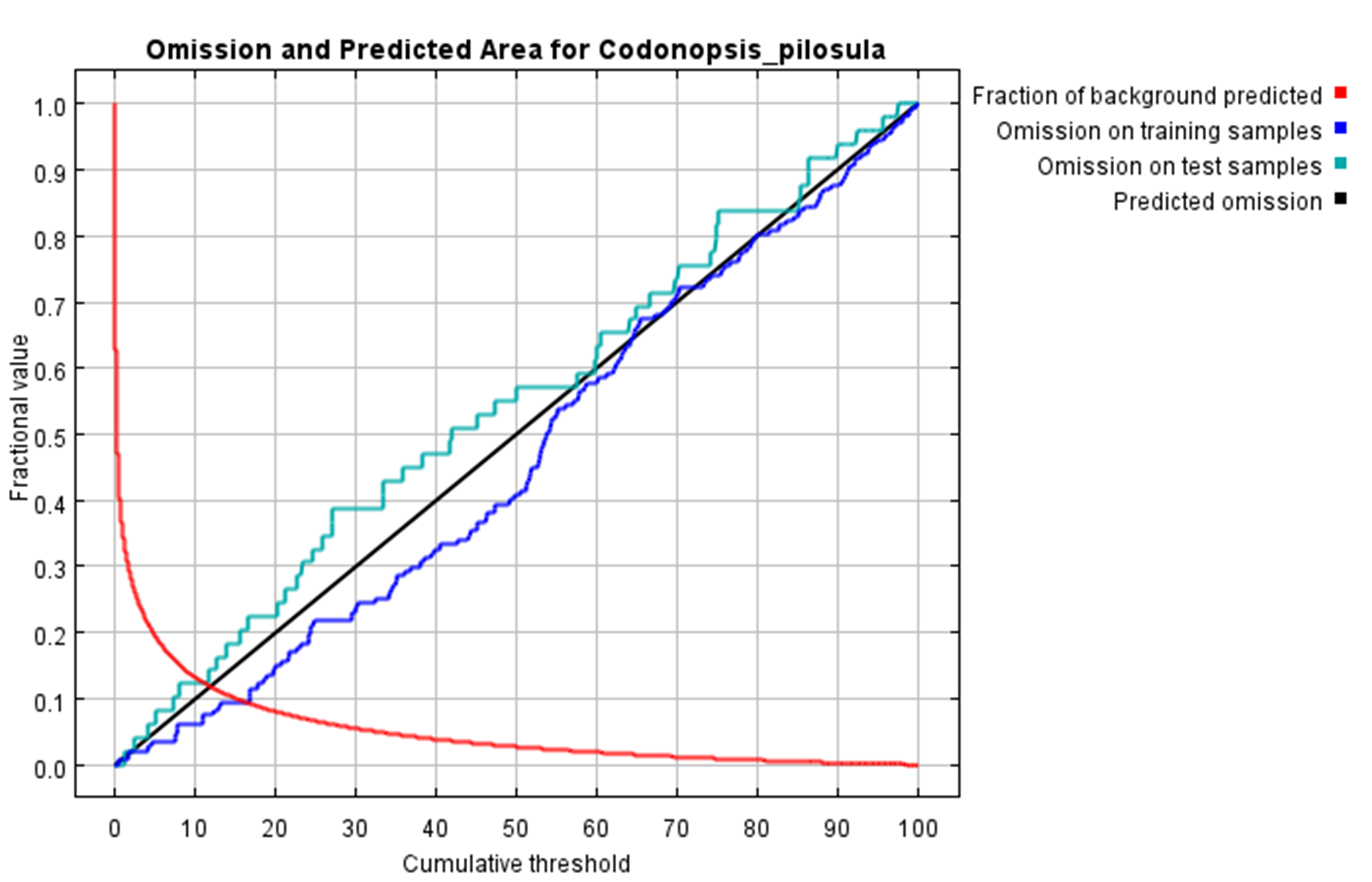
**

**Figure. S1.** The analysis omission of MaxEnt models for *Codonopsis pilosula.*





**Figure. S2.** The response curves of the ten ecological factors affecting the growth of *Codonopsis pilosula.*





**Figure. S3.** Comparison of functional factors concentration between different suitable habitats. (HS): samples collected from high suitable areas and (MS): samples collected from moderately suitable areas Note: Significant differences (*P* < 0.05) marked different lowercase letters.

**

**

**Figure. S4.** The spatial distribution map of the concentration of 22 functional factors in *Codonopsis Radix*(The maps were prepared by Zixia Wang and Yanjun Jia in ArcGIS Pro, https://www. esri. com/zh-cn/arcgis/products/arcgis-pro/resources).

**
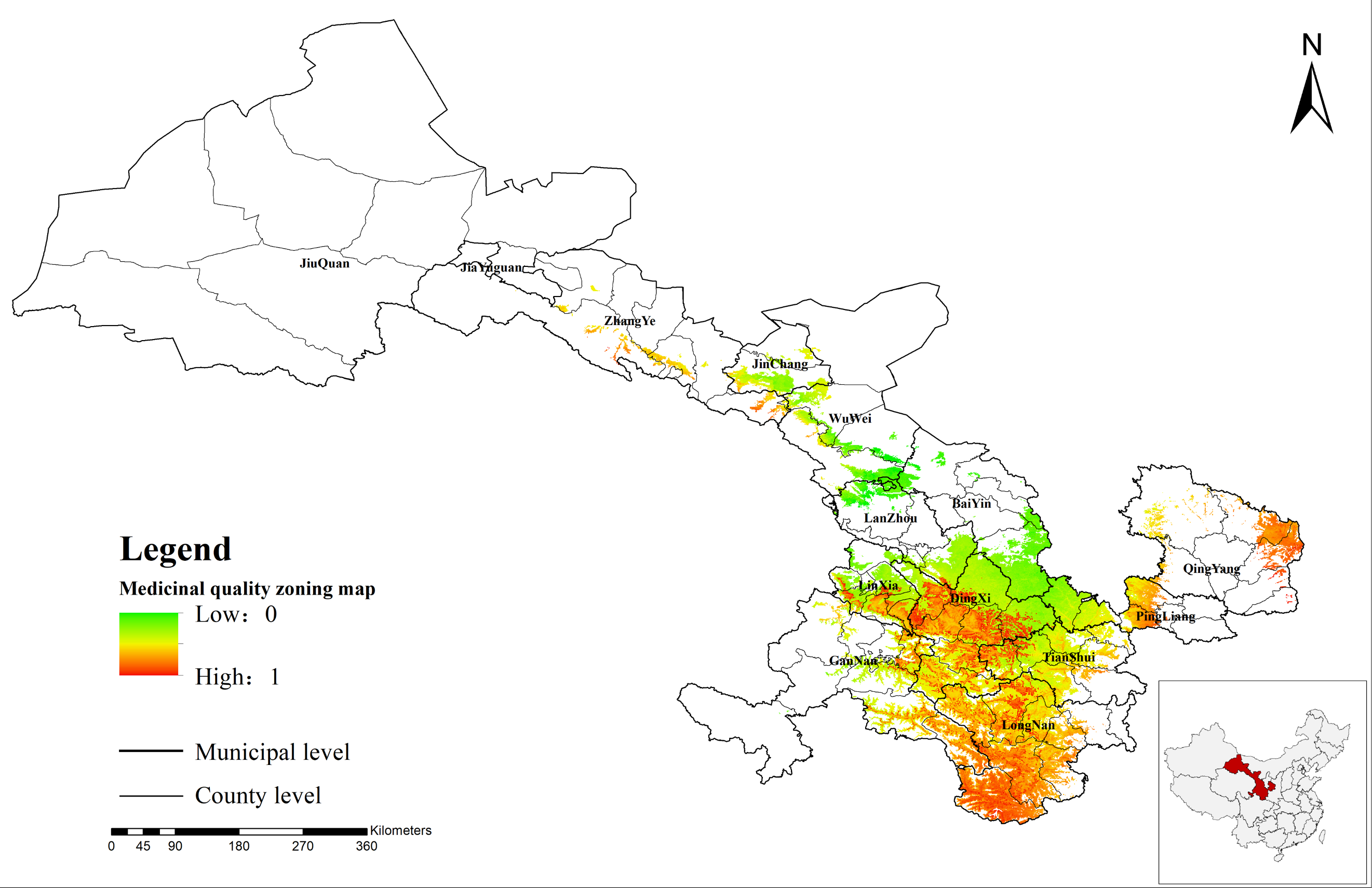
**

**Figure. S5.** The medicinal quality zoning map of *Codonopsis Radix* in Gansu Province(The maps were prepared by Zixia Wang and Yanjun Jia in ArcGIS Pro, https://www. esri. com/zh-cn/arcgis/products/arcgis-pro/resources).

**
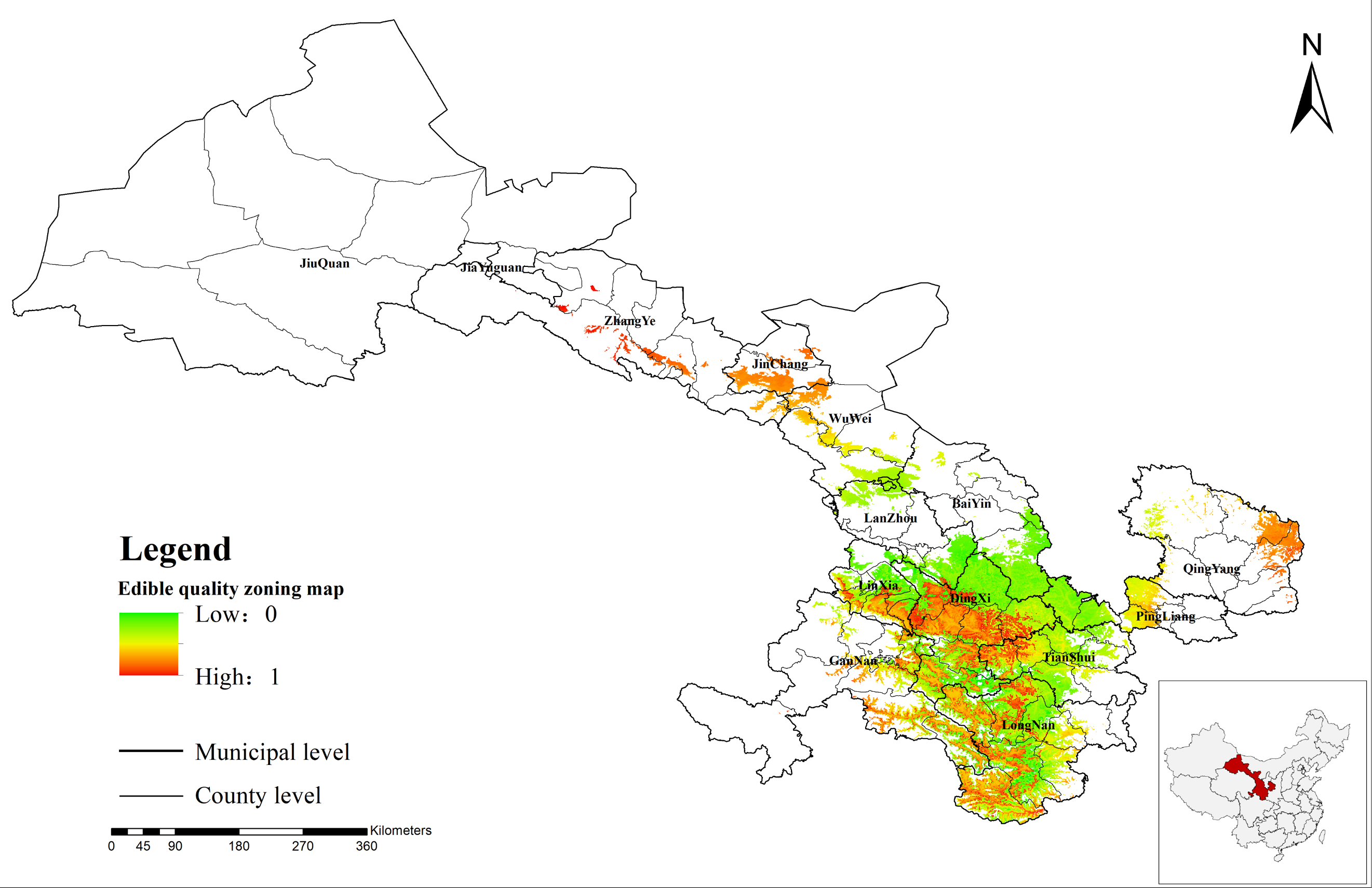
**

**Figure. S6.** The edible quality zoning map of *Codonopsis Radix* in Gansu Province(The maps were prepared by Zixia Wang and Yanjun Jia in ArcGIS Pro, https://www. esri. com/zh-cn/arcgis/products/arcgis-pro/resources).*.*
