# Supplementary material for: Study on environmental factors affecting the quality of codonopsis radix based on MaxEnt model and all-in-one functional factor
Source: Sci Rep. 2023 Nov 25;13:20726. doi: 10.1038/s41598-023-46546-6 (PMC10676394; doi:10.1038/s41598-023-46546-6)
Supplement: Supplementary file 2 — Supplementary Tables. [file 41598_2023_46546_MOESM2_ESM.docx]

**Study on environmental factors affecting the quality of *Codonopsis Radix* based on MaxEnt model and** **all-in-one** **functional factor**

**Zixia Wang^a#^，Yanjun Jia^a#^，Pengpeng Li^a^，Zhuoshi Tang^a^，Yina Guo^a^，Longxia Wen^a^，Huaqiao Yu^a^，Fang Cui^a,b,c,d^，Fangdi Hu^a,b,c,d*^**

^a^School of Pharmacy, Lanzhou University, Lanzhou, 730000, China

^b^State Key Laboratory of Applied Organic Chemistry, Lanzhou University, Lanzhou, 730000, China

^c^Codonopsis Radix Research Institute, Gansu Province, Lanzhou, 730000, China

^d^Codonopsis Radix Industrial Technology Engineering Research Center, Gansu Province, Lanzhou, 730000, China

^#^Yanjun Jia and Zixia Wang contributed equally to this work

^*^ Corresponding author.

E-mail: hufd@lzu.edu.cn; Fax: +860931 8915686; Tel: +860931 8911865/8911895

**Table S1.** Suitable value rang for main ecological factors of *Codonopsis pilosula.*

| Ecological factors | Percent contribution /% | Suitable range | Most suitable range |
| --- | --- | --- | --- |
| prec-10 | 37.3 | 27.0-60.0 mm | 33.0-55.2 mm |
| srad-10 | 57.4 | 0-11650 kJ·m^-2^·day^-1^ | 9350-11450 kJ·m^-2^·day^-1^ |
| prec-11 | 67.3 | 5.5-16.0 mm | 7.5-14.5 mm |
| bio-5 | 74.4 | 19.2-25.8 ℃ | 20.2-29.3 ℃ |
| prec-5 | 78.8 | 48.2-120.0 mm | 52.8-99.2 mm |
| prec-4 | 79.2 | 25.1-55.8 mm | 32.2-53.4 mm |
| su_sym90 | 84.9 | 1、2、4、5、7、8、9、11、13、16、35、50 | 1、4、5、7、9、13、16、35 |
| elev | 87 | 1600-2750 m | 1750-2650 m |
| awc_class | 90.5 | 1、2、3、6 | 1、2、3、6 |
| bio-14 | 92.7 | 0.5-5.0 mm | 1.0-4.3 mm |

Note: The su_Sym90 represents Soil Unit Symbol (FAO-90), and each number represents 1 (Fluvisols),2(Eutric Fluvisols),4(Dystric Fluvisols),5(Mollic Fluvisols),7(Thionic Fluvisols),8(Salic Fluviosls),9(Gleysols),11(Calcic Gleysols),13(Andic Gleysols),16(Thionic Gleysols),35(Vitric Andosols),50(Urbic Anthrosols).The awc_class represents AWC Range, and each number represents 1 (150mm/m),2(125mm/m),3(100mm/m),6(15mm/m).

**Table S2.** Sample information of *Codonopsis Radix* collected on site.

| NO. | Location | Longitude/°E | Latitude/°N |
| --- | --- | --- | --- |
| 1 | Liaojiazhai Village,Xinzhai Town,Weiyuan County,Dingxi City,Gansu Province,China | 104.16 | 35.37 |
| 2 | Kuanchuan Village,Xinzhai Town,Weiyuan County,Dingxi City,Gansu Province,China | 104.19 | 35.34 |
| 3 | Sanhe Village,Xinzhai Town,Weiyuan County,Dingxi City,Gansu Province,China | 104.19 | 35.31 |
| 4 | Quanwan Village,Xinzhai Town,Weiyuan County,Dingxi City,Gansu Province,China | 104.19 | 35.3 |
| 5 | Jianzicha Village,Xinzhai Town,Weiyuan County,Dingxi City,Gansu Province,China | 104.24 | 35.3 |
| 6 | Fengjiazhuang Village,Xinzhai Town,Weiyuan County,Dingxi City,Gansu Province,China | 104.21 | 35.28 |
| 7 | Dongpo Village,Xinzhai Town,Weiyuan County,Dingxi City,Gansu Province,China | 104.18 | 35.25 |
| 8 | Daping Village,Xinzhai Town,Weiyuan County,Dingxi City,Gansu Province,China | 104.22 | 35.25 |
| 9 | Liulin Village,Xinzhai Town,Weiyuan County,Dingxi City,Gansu Province,China | 104.16 | 35.23 |
| 10 | Kangjiashan Village,Xinzhai Town,Weiyuan County,Dingxi City,Gansu Province,China | 104.13 | 35.28 |
| 11 | Xinzhai Village,Xinzhai Town,Weiyuan County,Dingxi City,Gansu Province,China | 104.19 | 35.28 |
| 12 | Lijiawan Village,Xinzhai Town,Weiyuan County,Dingxi City,Gansu Province,China | 104.16 | 35.33 |
| 13 | Zhongzhai Village,Xinzhai Town,Weiyuan County,Dingxi City,Gansu Province,China | 104.25 | 35.24 |
| 14 | Xinzhai Town,Weiyuan County,Dingxi City,Gansu Province,China | 104.19 | 35.28 |
| 15 | Yanjiagou Village,Xinzhai Town,Weiyuan County,Dingxi City,Gansu Province,China | 104.23 | 35.28 |
| 16 | Malugou Village,Xinzhai Town,Weiyuan County,Dingxi City,Gansu Province,China | 104.48 | 35.16 |
| 17 | Lianmeng Village,Xinzhai Town,Weiyuan County,Dingxi City,Gansu Province,China | 104.22 | 35.21 |
| 18 | Fanjiawan Village,Qingping Town,Weiyuan County,Dingxi City,Gansu Province,China | 104.12 | 35.25 |
| 19 | Qingquan Village,Qingping Town,Weiyuan County,Dingxi City,Gansu Province,China | 104.14 | 35.23 |
| 20 | Guanshangen Village,Qingping Town,Weiyuan County,Dingxi City,Gansu Province,China | 104.13 | 35.21 |
| 21 | Wangjiachuan Village,Qingping Town,Weiyuan County,Dingxi City,Gansu Province,China | 104.11 | 35.19 |
| 22 | Majiayao Village,Qingyuan Town,Weiyuan County,Dingxi City,Gansu Province,China | 104.23 | 35.2 |
| 23 | Yaopo Village,Qingping Town,Weiyuan County,Dingxi City,Gansu Province,China | 104.12 | 35.22 |
| 24 | Lijiayao Village,Qingping Town,Weiyuan County,Dingxi City,Gansu Province,China | 104.1 | 35.24 |
| 25 | Panjiagou Village,Qingping Town,Weiyuan County,Dingxi City,Gansu Province,China | 104.12 | 35.27 |
| 26 | Gongjiagou Village,Qingping Town,Weiyuan County,Dingxi City,Gansu Province,China | 104.13 | 35.17 |
| 27 | Hongxian Village,Beizhai Town,Weiyuan County,Dingxi City,Gansu Province,China | 104.29 | 35.17 |
| 28 | Qisheng Village,Qingyuan Town,Weiyuan County,Dingxi City,Gansu Province,China | 104.23 | 35.17 |
| 29 | Liren Village,Qingyuan Town,Weiyuan County,Dingxi City,Gansu Province,China | 104.22 | 35.13 |
| 30 | Tianjiacha Village,Xinzhai Town,Weiyuan County,Dingxi City,Gansu Province,China | 104.16 | 35.34 |
| 31 | Cuijiahe Village,Qingyuan Town,Weiyuan County,Dingxi City,Gansu Province,China | 104.24 | 35.17 |
| 32 | Chiping Village,Qingyuan Town,Weiyuan County,Dingxi City,Gansu Province,China | 104.3 | 35.17 |
| 33 | Shangquan Village,Balipu Town,Lintao County,Dingxi City,Gansu Province,China | 103.95 | 35.48 |
| 34 | Nongmeng Village,Longmen Town,Lintao County,Dingxi City,Gansu Province,China | 103.96 | 35.38 |
| 35 | Yao Village,Yaodian Town,Lintao County,Dingxi City,Gansu Province,China | 104.06 | 35.3 |
| 36 | Nanguanping Village,Yujing Town,Lintao County,Dingxi City,Gansu Province,China | 103.84 | 35.28 |
| 37 | Ganguanping Village,Yujing Town,Lintao County,Dingxi City,Gansu Province,China | 103.84 | 35.28 |
| 38 | Wujia Village,Taoyang Town,Lintao County,Dingxi City,Gansu Province,China | 103.83 | 35.38 |
| 39 | Nongmeng Village,Longmen Town,Lintao County,Dingxi City,Gansu Province,China | 103.86 | 35.38 |
| 40 | Songjiaping Village,Yujing Town,Lintao County,Dingxi City,Gansu Province,China | 103.87 | 35.3 |
| 41 | Taoyang Town,Lintao County,Dingxi City,Gansu Province,China | 103.86 | 35.36 |
| 42 | Taoyang Town,Lintao County,Dingxi City,Gansu Province,China | 103.86 | 35.36 |
| 43 | Zhaojiaying Village,Dexing Town,Longxi County,Dingxi City,Gansu Province,China | 104.46 | 35.2 |
| 44 | Shangshanzhuang Village,Dexing Town,Longxi County,Dingxi City,Gansu Province,China | 104.46 | 35.18 |
| 45 | Zhongjiamen Village,Shuangquan Town,Longxi County,Dingxi City,Gansu Province,China | 104.41 | 35.18 |
| 46 | Dongjiabao Village,Shuangquan Town,Longxi County,Dingxi City,Gansu Province,China | 104.42 | 35.14 |
| 47 | Pangjiacha Village,Fuxing Town,Longxi County,Dingxi City,Gansu Province,China | 104.55 | 35.19 |
| 48 | Houjiacha Village,Fuxing Town,Longxi County,Dingxi City,Gansu Province,China | 104.55 | 35.19 |
| 49 | Gaoleng Village,Fuxing Town,Longxi County,Dingxi City,Gansu Province,China | 104.58 | 35.09 |
| 50 | Weijiawan Village,Biyan Town,Longxi County,Dingxi City,Gansu Province,China | 104.42 | 35.03 |
| 51 | Longchuan Village,Biyan Town,Longxi County,Dingxi City,Gansu Province,China | 104.48 | 35.02 |
| 52 | Zhongchuan Village,Caizi Town,Longxi County,Dingxi City,Gansu Province,China | 104.41 | 34.98 |
| 53 | Zhongchuan Village,Caizi Town,Longxi County,Dingxi City,Gansu Province,China | 104.41 | 34.98 |
| 54 | Fanjiamen Village,Caizi Town,Longxi County,Dingxi City,Gansu Province,China | 104.39 | 34.96 |
| 55 | Jinwan Village,Gongchang Town,Longxi County,Dingxi City,Gansu Province,China | 104.55 | 34.93 |
| 56 | Jinwan Village,Gongchang Town,Longxi County,Dingxi City,Gansu Province,China | 104.55 | 34.93 |
| 57 | Baiyanglin Village,Gongchang Town,Longxi County,Dingxi City,Gansu Province,China | 104.56 | 34.96 |
| 58 | Sanheqiaomen Village,Wenfeng Town,Longxi County,Dingxi City,Gansu Province,China | 104.74 | 34.92 |
| 59 | Baozi Village,Yongji Town,Longxi County,Dingxi City,Gansu Province,China | 104.87 | 34.89 |
| 60 | Yaojiagou Village,Yongji Town,Longxi County,Dingxi City,Gansu Province,China | 104.9 | 34.91 |
| 61 | Lannigou Village,Yongji Town,Longxi County,Dingxi City,Gansu Province,China | 104.89 | 34.9 |
| 62 | Yuanyi Village,Gongchang Town,Longxi County,Dingxi City,Gansu Province,China | 104.62 | 35.02 |
| 63 | Yuanyi Village,Gongchang Town,Longxi County,Dingxi City,Gansu Province,China | 104.62 | 35.02 |
| 64 | Jianzi Village,Jinzhong Town,Zhang County,Dingxi City,Gansu Province,China | 104.12 | 34.82 |
| 65 | Xinlian Village,Dacaotan Town,Zhang County,Dingxi City,Gansu Province,China | 104.26 | 34.77 |
| 66 | Chenjiazui,Maquan Town,Zhang County,Dingxi City,Gansu Province,China | 104.53 | 34.72 |
| 67 | Jinzhong Town,Zhang County,Dingxi City,Gansu Province,China | 104.15 | 34.81 |
| 68 | Caochangjie Village,Dacaotan Town,Zhang County,Dingxi City,Gansu Province,China | 104.19 | 34.75 |
| 69 | Maquan Town,Zhang County,Dingxi City,Gansu Province,China | 104.46 | 34.73 |
| 70 | Zhuoping Village,Weixin Town,Min County,Dingxi City,Gansu Province,China | 103.92 | 34.65 |
| 71 | Zhama Village,Zhongzhai Town,Min County,Dingxi City,Gansu Province,China | 103.96 | 34.64 |
| 72 | Donggou Village,Xizhai Town,Min County,Dingxi City,Gansu Province,China | 103.83 | 34.5 |
| 73 | Shandixia Village,Qingshui Town,Min County,Dingxi City,Gansu Province,China | 103.98 | 34.42 |
| 74 | Lamei Village,Qingshui Town,Min County,Dingxi City,Gansu Province,China | 103.93 | 34.44 |
| 75 | Tangjiachuan Village,Xijiang Town,Min County,Dingxi City,Gansu Province,China | 104.02 | 34.55 |
| 76 | Tiechi Village,Xijiang Town,Min County,Dingxi City,Gansu Province,China | 104.04 | 34.54 |
| 77 | Ningning Village,Meichuan Town,Min County,Dingxi City,Gansu Province,China | 104.11 | 34.53 |
| 78 | Yongguang Village,Meichuan Town,Min County,Dingxi City,Gansu Province,China | 104.15 | 34.51 |
| 79 | Sanshilipu Village,Shili Town,Min County,Dingxi City,Gansu Province,China | 103.89 | 34.45 |
| 80 | Leijia Village,Shili Town,Min County,Dingxi City,Gansu Province,China | 103.99 | 34.42 |
| 81 | Baitasi Village,Minyang Town,Min County,Dingxi City,Gansu Province,China | 104.06 | 34.44 |
| 82 | Dongzhao Village,Chenjiao Town,Min County,Dingxi City,Gansu Province,China | 104.05 | 34.43 |
| 83 | Yangpo Village,Chafu Town,Min County,Dingxi City,Gansu Province,China | 104.09 | 34.49 |
| 84 | Puli Village,Chafu Town,Min County,Dingxi City,Gansu Province,China | 104.1 | 34.48 |
| 85 | Qiaojiagou Village,Hetuo Town,Min County,Dingxi City,Gansu Province,China | 104.22 | 34.48 |
| 86 | Shijiatai Village,Hetuo Town,Min County,Dingxi City,Gansu Province,China | 104.28 | 34.42 |
| 87 | Luyuan Village,Mazichuan Town,Min County,Dingxi City,Gansu Province,China | 104.09 | 34.3 |
| 88 | Xuanwo Village,Mazichuan Town,Min County,Dingxi City,Gansu Province,China | 104.05 | 34.25 |
| 89 | Awu Town,Tanchang County,Longnan City,Gansu Province,China | 104.15 | 34.29 |
| 90 | Hada Village,Awu Town,Tanchang County,Longnan City,Gansu Province,China | 104.16 | 34.28 |
| 91 | Pangjia Town,Tanchang County,Longnan City,Gansu Province,China | 104.29 | 34.29 |
| 92 | Hadapu Town,Tanchang County,Longnan City,Gansu Province,China | 104.23 | 34.23 |
| 93 | Wanliba Village,Haoti Town,Tanchang County,Longnan City,Gansu Province,China | 104.75 | 33.97 |
| 94 | Bielong Village,Awu Town,Tanchang County,Longnan City,Gansu Province,China | 104.15 | 34.3 |
| 95 | Pashi Village,Awu Town,Tanchang County,Longnan City,Gansu Province,China | 104.59 | 34.67 |
| 96 | Xidie Village,Awu Town,Tanchang County,Longnan City,Gansu Province,China | 104.16 | 34.27 |
| 97 | Xinzhai Village,Hadapu Town,Tanchang County,Longnan City,Gansu Province,China | 104.59 | 34.67 |
| 98 | Jinbuchuan Village,Hadapu Town,Tanchang County,Longnan City,Gansu Province,China | 104.23 | 34.23 |
| 99 | Hezang Village,Hadapu Town,Tanchang County,Longnan City,Gansu Province,China | 104.24 | 34.27 |
| 100 | Songzha Village,Pangjia Town,Tanchang County,Longnan City,Gansu Province,China | 104.27 | 34.31 |
| 101 | Taer Village,Pangjia Town,Tanchang County,Longnan City,Gansu Province,China | 104.28 | 34.3 |
| 102 | Xujia Village,Pangjia Town,Tanchang County,Longnan City,Gansu Province,China | 104.26 | 34.31 |
| 103 | Caijia Village,Lichuan Town,Tanchang County,Longnan City,Gansu Province,China | 104.31 | 34.24 |
| 104 | Zhongbaozi Village,Lichuan Town,Tanchang County,Longnan City,Gansu Province,China | 104.29 | 34.23 |
| 105 | Lasha Village,Lichuan Town,Tanchang County,Longnan City,Gansu Province,China | 104.34 | 34.27 |
| 106 | Shimen Village,Bali Town,Tanchang County,Longnan City,Gansu Province,China | 104.34 | 34.3 |
| 107 | Bulong Village,Muer Town,Tanchang County,Longnan City,Gansu Province,China | 104.34 | 34.27 |
| 108 | Hanyuan Village,Hanyuan Town,Tanchang County,Longnan City,Gansu Province,China | 104.68 | 33.96 |
| 109 | Qiqi Village,Dianzi Town,Lintan County,Gannan Tibetan Autonomous Prefecture,Gansu Province,China | 103.67 | 34.63 |
| 110 | Zongzhai Village,Taobin Town,Lintan County,Gannan Tibetan Autonomous Prefecture,Gansu Province,China | 103.69 | 34.55 |
| 111 | Shiqi Village,Zongzhai Town,Lintan County,Gannan Tibetan Autonomous Prefecture,Gansu Province,China | 103.69 | 34.54 |
| 112 | Zhangqi Village,Xinchen Town,Lintan County,Gannan Tibetan Autonomous Prefecture,Gansu Province,China | 103.63 | 34.63 |
| 113 | Gudiba Village,Boyu Town,Zhouqu County,Gannan Tibetan Autonomous Prefecture,Gansu Province,China | 104.33 | 33.42 |
| 114 | Xiyan Village,Liping Town,Wenxian County,Longnan City,Gansu Province,China | 104.87 | 33.2 |
| 115 | Liujiawan Village,Qiaotou Town,Wenxian County,Longnan City,Gansu Province,China | 104.72 | 33.11 |
| 116 | Songping Village,Zhongzhai Town,Wenxian County,Longnan City,Gansu Province,China | 104.47 | 33.28 |
| 117 | Jijiabao Village,Baoziba Town,Wenxian County,Longnan City,Gansu Province,China | 104.65 | 33.12 |
| 118 | Baoziping Village,Baoziba Town,Wenxian County,Longnan City,Gansu Province,China | 104.66 | 33.13 |
| 119 | Deshengqian Village,Baoziba Town,Wenxian County,Longnan City,Gansu Province,China | 104.64 | 33.12 |
| 120 | Shangdanbao Village,Danbao Town,Wenxian County,Longnan City,Gansu Province,China | 104.78 | 32.86 |
| 121 | Shangdanbao Village,Danbao Town,Wenxian County,Longnan City,Gansu Province,China | 104.54 | 32.86 |
| 122 | Liziba Village,Tielou Tibetan Town,Wenxian County,Longnan City,Gansu Province,China | 104.38 | 32.9 |
| 123 | Haxigou Village,Zhongzhai Town,Wenxian County,Longnan City,Gansu Province,China | 104.38 | 33.25 |
| 124 | Zhongzhai Village,Zhongzhai Town,Wenxian County,Longnan City,Gansu Province,China | 104.42 | 33.19 |
| 125 | Xigoushan Village,Koutouba Town,Wenxian County,Longnan City,Gansu Province,China | 104.85 | 32.96 |
| 126 | Doujiawan Village,Koutouba Town,Wenxian County,Longnan City,Gansu Province,China | 104.96 | 33 |
| 127 | Fengyuanshan Village,Shangde Town,Wenxian County,Longnan City,Gansu Province,China | 104.77 | 32.95 |
| 128 | Tianchi Village,Tianchi Town,Wenxian County,Longnan City,Gansu Province,China | 104.41 | 33.24 |
| 129 | Tianchi Village,Tianchi Town,Wenxian County,Longnan City,Gansu Province,China | 104.41 | 33.24 |
| 130 | Tianchi Village,Tianchi Town,Wenxian County,Longnan City,Gansu Province,China | 104.41 | 33.24 |
| 131 | Tianchi Village,Tianchi Town,Wenxian County,Longnan City,Gansu Province,China | 104.41 | 33.24 |
| 132 | Zhangjiawan Village,Qiaotou Town,Wenxian County,Longnan City,Gansu Province,China | 104.78 | 33.05 |
| 133 | Jijiabao Village,Baoziba Town,Wenxian County,Longnan City,Gansu Province,China | 104.65 | 33.12 |
| 134 | Yangzhishan Village,Zhongzhai Town,Wenxian County,Longnan City,Gansu Province,China | 104.41 | 33.17 |

**Table S3.** The potential suitable areas of of *Codonopsis pilosula* in Gansu Province.

| Suitable type | Area/×10^4^ km^2^ | Proportion/% |
| --- | --- | --- |
| Unsuitable area | 34.78 | 81.00 |
| Low suitable area | 4.25 | 9.90 |
| Moderate suitable area | 2.12 | 4.94 |
| High suitable area | 1.78 | 4.15 |

**Table S4.** Corresponding eigenvalues and contribution rates of principal components.

| Principal component | eigenvalue | contribution rate（%） | Cumulative contribution rate（%） |
| --- | --- | --- | --- |
| PC1 | 8.938 | 49.690 | 49.690 |
| PC2 | 2.810 | 18.050 | 67.740 |
| PC3 | 2.046 | 10.232 | 77.973 |
| PC4 | 1.443 | 7.214 | 85.187 |
| PC5 | 1.041 | 5.207 | 90.394 |

**Table S5.** Factor load matrix.

| common peak | PC1 | PC2 | PC3 | PC4 | PC5 |
| --- | --- | --- | --- | --- | --- |
| 1 | 0.913 | 0.374 | -0.054 | -0.331 | 0.295 |
| 2 | 0.230 | 0.487 | -0.329 | 0.001 | -0.344 |
| 3 | 0.362 | -0.130 | 0.505 | 0.235 | 0.100 |
| 4 | 0.887 | -0.517 | 0.060 | 0.030 | 0.193 |
| 5 | 0.445 | 0.456 | 0.131 | 0.893 | -0.025 |
| 6 | 0.310 | -0.227 | 0.915 | 0.052 | -0.089 |
| 7 | 0.628 | -0.169 | 0.391 | 0.044 | 0.398 |
| 8 | 0.293 | -0.370 | -0.242 | -0.184 | 0.161 |
| 9 | 0.934 | 0.443 | 0.450 | -0.010 | 0.041 |
| 10 | 0.551 | -0.044 | 0.092 | 0.455 | -0.898 |
| 11 | 0.852 | 0.067 | 0.138 | 0.018 | 0.028 |
| 12 | 0.571 | 0.866 | -0.103 | -0.405 | 0.057 |
| 13 | 0.621 | 0.151 | 0.077 | -0.416 | -0.075 |
| 14 | 0.884 | -0.442 | -0.318 | -0.064 | -0.065 |
| 15 | 0.949 | -0.177 | -0.346 | -0.095 | -0.445 |
| 16 | 0.510 | 0.056 | 0.204 | -0.147 | -0.445 |
| 17 | 0.547 | -0.201 | -0.363 | 0.024 | 0.165 |
| 18 | 0.414 | -0.064 | -0.422 | 0.125 | 0.126 |
| 19 | 0.258 | 0.950 | -0.176 | 0.313 | 0.224 |
| 20 | 0.214 | 0.227 | -0.374 | 0.244 | 0.309 |

**Table S6.** Habitat suitability grade of *Codonopsis Radix* sample distribution points.

| NO. | Location | Suitability | Habitat suitability | Abbreviation |
| --- | --- | --- | --- | --- |
| 1 | Liaojiazhai Village,Xinzhai Town,Weiyuan County,Dingxi City,Gansu Province,China | 0.89 | high suitable area | HS |
| 2 | Kuanchuan Village,Xinzhai Town,Weiyuan County,Dingxi City,Gansu Province,China | 0.87 | high suitable area | HS |
| 3 | Sanhe Village,Xinzhai Town,Weiyuan County,Dingxi City,Gansu Province,China | 0.93 | high suitable area | HS |
| 4 | Quanwan Village,Xinzhai Town,Weiyuan County,Dingxi City,Gansu Province,China | 0.85 | high suitable area | HS |
| 5 | Jianzicha Village,Xinzhai Town,Weiyuan County,Dingxi City,Gansu Province,China | 0.88 | high suitable area | HS |
| 6 | Fengjiazhuang Village,Xinzhai Town,Weiyuan County,Dingxi City,Gansu Province,China | 0.94 | high suitable area | HS |
| 7 | Dongpo Village,Xinzhai Town,Weiyuan County,Dingxi City,Gansu Province,China | 0.90 | high suitable area | HS |
| 8 | Daping Village,Xinzhai Town,Weiyuan County,Dingxi City,Gansu Province,China | 0.85 | high suitable area | HS |
| 9 | Liulin Village,Xinzhai Town,Weiyuan County,Dingxi City,Gansu Province,China | 0.86 | high suitable area | HS |
| 10 | Kangjiashan Village,Xinzhai Town,Weiyuan County,Dingxi City,Gansu Province,China | 0.93 | high suitable area | HS |
| 11 | Xinzhai Village,Xinzhai Town,Weiyuan County,Dingxi City,Gansu Province,China | 0.80 | high suitable area | HS |
| 12 | Lijiawan Village,Xinzhai Town,Weiyuan County,Dingxi City,Gansu Province,China | 0.77 | high suitable area | HS |
| 13 | Zhongzhai Village,Xinzhai Town,Weiyuan County,Dingxi City,Gansu Province,China | 0.94 | high suitable area | HS |
| 14 | Xinzhai Town,Weiyuan County,Dingxi City,Gansu Province,China | 0.80 | high suitable area | HS |
| 15 | Yanjiagou Village,Xinzhai Town,Weiyuan County,Dingxi City,Gansu Province,China | 0.90 | high suitable area | HS |
| 16 | Malugou Village,Xinzhai Town,Weiyuan County,Dingxi City,Gansu Province,China | 0.82 | high suitable area | HS |
| 17 | Lianmeng Village,Xinzhai Town,Weiyuan County,Dingxi City,Gansu Province,China | 0.91 | high suitable area | HS |
| 18 | Fanjiawan Village,Qingping Town,Weiyuan County,Dingxi City,Gansu Province,China | 0.91 | high suitable area | HS |
| 19 | Qingquan Village,Qingping Town,Weiyuan County,Dingxi City,Gansu Province,China | 0.84 | high suitable area | HS |
| 20 | Guanshangen Village,Qingping Town,Weiyuan County,Dingxi City,Gansu Province,China | 0.95 | high suitable area | HS |
| 21 | Wangjiachuan Village,Qingping Town,Weiyuan County,Dingxi City,Gansu Province,China | 0.95 | high suitable area | HS |
| 22 | Majiayao Village,Qingyuan Town,Weiyuan County,Dingxi City,Gansu Province,China | 0.97 | high suitable area | HS |
| 23 | Yaopo Village,Qingping Town,Weiyuan County,Dingxi City,Gansu Province,China | 0.95 | high suitable area | HS |
| 24 | Lijiayao Village,Qingping Town,Weiyuan County,Dingxi City,Gansu Province,China | 0.93 | high suitable area | HS |
| 25 | Panjiagou Village,Qingping Town,Weiyuan County,Dingxi City,Gansu Province,China | 0.90 | high suitable area | HS |
| 26 | Gongjiagou Village,Qingping Town,Weiyuan County,Dingxi City,Gansu Province,China | 0.78 | high suitable area | HS |
| 27 | Hongxian Village,Beizhai Town,Weiyuan County,Dingxi City,Gansu Province,China | 0.90 | high suitable area | HS |
| 28 | Qisheng Village,Qingyuan Town,Weiyuan County,Dingxi City,Gansu Province,China | 0.95 | high suitable area | HS |
| 29 | Liren Village,Qingyuan Town,Weiyuan County,Dingxi City,Gansu Province,China | 0.98 | high suitable area | HS |
| 30 | Tianjiacha Village,Xinzhai Town,Weiyuan County,Dingxi City,Gansu Province,China | 0.79 | high suitable area | HS |
| 31 | Cuijiahe Village,Qingyuan Town,Weiyuan County,Dingxi City,Gansu Province,China | 0.95 | high suitable area | HS |
| 32 | Chiping Village,Qingyuan Town,Weiyuan County,Dingxi City,Gansu Province,China | 0.85 | high suitable area | HS |
| 33 | Shangquan Village,Balipu Town,Lintao County,Dingxi City,Gansu Province,China | 0.59 | moderate suitable area | MS |
| 34 | Nongmeng Village,Longmen Town,Lintao County,Dingxi City,Gansu Province,China | 0.71 | high suitable area | HS |
| 35 | Yao Village,Yaodian Town,Lintao County,Dingxi City,Gansu Province,China | 0.87 | high suitable area | HS |
| 36 | Nanguanping Village,Yujing Town,Lintao County,Dingxi City,Gansu Province,China | 0.64 | high suitable area | HS |
| 37 | Ganguanping Village,Yujing Town,Lintao County,Dingxi City,Gansu Province,China | 0.64 | high suitable area | HS |
| 38 | Wujia Village,Taoyang Town,Lintao County,Dingxi City,Gansu Province,China | 0.56 | moderate suitable area | MS |
| 39 | Nongmeng Village,Longmen Town,Lintao County,Dingxi City,Gansu Province,China | 0.58 | moderate suitable area | MS |
| 40 | Songjiaping Village,Yujing Town,Lintao County,Dingxi City,Gansu Province,China | 0.74 | high suitable area | HS |
| 41 | Taoyang Town,Lintao County,Dingxi City,Gansu Province,China | 0.46 | moderate suitable area | MS |
| 42 | Taoyang Town,Lintao County,Dingxi City,Gansu Province,China | 0.46 | moderate suitable area | MS |
| 43 | Zhaojiaying Village,Dexing Town,Longxi County,Dingxi City,Gansu Province,China | 0.81 | high suitable area | HS |
| 44 | Shangshanzhuang Village,Dexing Town,Longxi County,Dingxi City,Gansu Province,China | 0.79 | high suitable area | HS |
| 45 | Zhongjiamen Village,Shuangquan Town,Longxi County,Dingxi City,Gansu Province,China | 0.86 | high suitable area | HS |
| 46 | Dongjiabao Village,Shuangquan Town,Longxi County,Dingxi City,Gansu Province,China | 0.97 | high suitable area | HS |
| 47 | Pangjiacha Village,Fuxing Town,Longxi County,Dingxi City,Gansu Province,China | 0.90 | high suitable area | HS |
| 48 | Houjiacha Village,Fuxing Town,Longxi County,Dingxi City,Gansu Province,China | 0.90 | high suitable area | HS |
| 49 | Gaoleng Village,Fuxing Town,Longxi County,Dingxi City,Gansu Province,China | 0.78 | high suitable area | HS |
| 50 | Weijiawan Village,Biyan Town,Longxi County,Dingxi City,Gansu Province,China | 0.70 | high suitable area | HS |
| 51 | Longchuan Village,Biyan Town,Longxi County,Dingxi City,Gansu Province,China | 0.82 | high suitable area | HS |
| 52 | Zhongchuan Village,Caizi Town,Longxi County,Dingxi City,Gansu Province,China | 0.72 | high suitable area | HS |
| 53 | Zhongchuan Village,Caizi Town,Longxi County,Dingxi City,Gansu Province,China | 0.72 | high suitable area | HS |
| 54 | Fanjiamen Village,Caizi Town,Longxi County,Dingxi City,Gansu Province,China | 0.78 | high suitable area | HS |
| 55 | Jinwan Village,Gongchang Town,Longxi County,Dingxi City,Gansu Province,China | 0.60 | moderate suitable area | MS |
| 56 | Jinwan Village,Gongchang Town,Longxi County,Dingxi City,Gansu Province,China | 0.60 | moderate suitable area | MS |
| 57 | Baiyanglin Village,Gongchang Town,Longxi County,Dingxi City,Gansu Province,China | 0.69 | high suitable area | HS |
| 58 | Sanheqiaomen Village,Wenfeng Town,Longxi County,Dingxi City,Gansu Province,China | 0.96 | high suitable area | HS |
| 59 | Baozi Village,Yongji Town,Longxi County,Dingxi City,Gansu Province,China | 0.55 | moderate suitable area | MS |
| 60 | Yaojiagou Village,Yongji Town,Longxi County,Dingxi City,Gansu Province,China | 0.63 | high suitable area | HS |
| 61 | Lannigou Village,Yongji Town,Longxi County,Dingxi City,Gansu Province,China | 0.60 | moderate suitable area | MS |
| 62 | Yuanyi Village,Gongchang Town,Longxi County,Dingxi City,Gansu Province,China | 0.89 | high suitable area | HS |
| 63 | Yuanyi Village,Gongchang Town,Longxi County,Dingxi City,Gansu Province,China | 0.89 | high suitable area | HS |
| 64 | Jianzi Village,Jinzhong Town,Zhang County,Dingxi City,Gansu Province,China | 0.99 | high suitable area | HS |
| 65 | Xinlian Village,Dacaotan Town,Zhang County,Dingxi City,Gansu Province,China | 0.70 | high suitable area | HS |
| 66 | Chenjiazui,Maquan Town,Zhang County,Dingxi City,Gansu Province,China | 0.65 | high suitable area | HS |
| 67 | Jinzhong Town,Zhang County,Dingxi City,Gansu Province,China | 0.65 | high suitable area | HS |
| 68 | Caochangjie Village,Dacaotan Town,Zhang County,Dingxi City,Gansu Province,China | 0.97 | high suitable area | HS |
| 69 | Maquan Town,Zhang County,Dingxi City,Gansu Province,China | 0.92 | high suitable area | HS |
| 70 | Zhuoping Village,Weixin Town,Min County,Dingxi City,Gansu Province,China | 0.97 | high suitable area | HS |
| 71 | Zhama Village,Zhongzhai Town,Min County,Dingxi City,Gansu Province,China | 0.97 | high suitable area | HS |
| 72 | Donggou Village,Xizhai Town,Min County,Dingxi City,Gansu Province,China | 0.90 | high suitable area | HS |
| 73 | Shandixia Village,Qingshui Town,Min County,Dingxi City,Gansu Province,China | 0.99 | high suitable area | HS |
| 74 | Lamei Village,Qingshui Town,Min County,Dingxi City,Gansu Province,China | 0.90 | high suitable area | HS |
| 75 | Tangjiachuan Village,Xijiang Town,Min County,Dingxi City,Gansu Province,China | 1.00 | high suitable area | HS |
| 76 | Tiechi Village,Xijiang Town,Min County,Dingxi City,Gansu Province,China | 1.00 | high suitable area | HS |
| 77 | Ningning Village,Meichuan Town,Min County,Dingxi City,Gansu Province,China | 0.88 | high suitable area | HS |
| 78 | Yongguang Village,Meichuan Town,Min County,Dingxi City,Gansu Province,China | 0.90 | high suitable area | HS |
| 79 | Sanshilipu Village,Shili Town,Min County,Dingxi City,Gansu Province,China | 1.00 | high suitable area | HS |
| 80 | Leijia Village,Shili Town,Min County,Dingxi City,Gansu Province,China | 0.98 | high suitable area | HS |
| 81 | Baitasi Village,Minyang Town,Min County,Dingxi City,Gansu Province,China | 0.97 | high suitable area | HS |
| 82 | Dongzhao Village,Chenjiao Town,Min County,Dingxi City,Gansu Province,China | 0.98 | high suitable area | HS |
| 83 | Yangpo Village,Chafu Town,Min County,Dingxi City,Gansu Province,China | 0.97 | high suitable area | HS |
| 84 | Puli Village,Chafu Town,Min County,Dingxi City,Gansu Province,China | 0.96 | high suitable area | HS |
| 85 | Qiaojiagou Village,Hetuo Town,Min County,Dingxi City,Gansu Province,China | 0.43 | moderate suitable area | MS |
| 86 | Shijiatai Village,Hetuo Town,Min County,Dingxi City,Gansu Province,China | 0.47 | moderate suitable area | MS |
| 87 | Luyuan Village,Mazichuan Town,Min County,Dingxi City,Gansu Province,China | 0.56 | moderate suitable area | MS |
| 88 | Xuanwo Village,Mazichuan Town,Min County,Dingxi City,Gansu Province,China | 0.45 | moderate suitable area | MS |
| 89 | Awu Town,Tanchang County,Longnan City,Gansu Province,China | 0.92 | high suitable area | HS |
| 90 | Hada Village,Awu Town,Tanchang County,Longnan City,Gansu Province,China | 0.96 | high suitable area | HS |
| 91 | Pangjia Town,Tanchang County,Longnan City,Gansu Province,China | 0.99 | high suitable area | HS |
| 92 | Hadapu Town,Tanchang County,Longnan City,Gansu Province,China | 0.99 | high suitable area | HS |
| 93 | Wanliba Village,Haoti Town,Tanchang County,Longnan City,Gansu Province,China | 0.73 | high suitable area | HS |
| 94 | Bielong Village,Awu Town,Tanchang County,Longnan City,Gansu Province,China | 0.90 | high suitable area | HS |
| 95 | Pashi Village,Awu Town,Tanchang County,Longnan City,Gansu Province,China | 0.47 | moderate suitable area | MS |
| 96 | Xidie Village,Awu Town,Tanchang County,Longnan City,Gansu Province,China | 0.97 | high suitable area | HS |
| 97 | Xinzhai Village,Hadapu Town,Tanchang County,Longnan City,Gansu Province,China | 0.47 | moderate suitable area | MS |
| 98 | Jinbuchuan Village,Hadapu Town,Tanchang County,Longnan City,Gansu Province,China | 0.99 | high suitable area | HS |
| 99 | Hezang Village,Hadapu Town,Tanchang County,Longnan City,Gansu Province,China | 0.99 | high suitable area | HS |
| 100 | Songzha Village,Pangjia Town,Tanchang County,Longnan City,Gansu Province,China | 0.97 | high suitable area | HS |
| 101 | Taer Village,Pangjia Town,Tanchang County,Longnan City,Gansu Province,China | 0.99 | high suitable area | HS |
| 102 | Xujia Village,Pangjia Town,Tanchang County,Longnan City,Gansu Province,China | 0.98 | high suitable area | HS |
| 103 | Caijia Village,Lichuan Town,Tanchang County,Longnan City,Gansu Province,China | 1.00 | high suitable area | HS |
| 104 | Zhongbaozi Village,Lichuan Town,Tanchang County,Longnan City,Gansu Province,China | 0.98 | high suitable area | HS |
| 105 | Lasha Village,Lichuan Town,Tanchang County,Longnan City,Gansu Province,China | 0.97 | high suitable area | HS |
| 106 | Shimen Village,Bali Town,Tanchang County,Longnan City,Gansu Province,China | 0.99 | high suitable area | HS |
| 107 | Bulong Village,Muer Town,Tanchang County,Longnan City,Gansu Province,China | 0.97 | high suitable area | HS |
| 108 | Hanyuan Village,Hanyuan Town,Tanchang County,Longnan City,Gansu Province,China | 0.97 | high suitable area | HS |
| 109 | Qiqi Village,Dianzi Town,Lintan County,Gannan Tibetan Autonomous Prefecture,Gansu Province,China | 0.85 | high suitable area | HS |
| 110 | Zongzhai Village,Taobin Town,Lintan County,Gannan Tibetan Autonomous Prefecture,Gansu Province,China | 0.90 | high suitable area | HS |
| 111 | Shiqi Village,Zongzhai Town,Lintan County,Gannan Tibetan Autonomous Prefecture,Gansu Province,China | 0.86 | high suitable area | HS |
| 112 | Zhangqi Village,Xinchen Town,Lintan County,Gannan Tibetan Autonomous Prefecture,Gansu Province,China | 0.97 | high suitable area | HS |
| 113 | Gudiba Village,Boyu Town,Zhouqu County,Gannan Tibetan Autonomous Prefecture,Gansu Province,China | 0.05 | Unsuitable area | US |
| 114 | Xiyan Village,Liping Town,Wenxian County,Longnan City,Gansu Province,China | 0.80 | high suitable area | HS |
| 115 | Liujiawan Village,Qiaotou Town,Wenxian County,Longnan City,Gansu Province,China | 0.87 | high suitable area | HS |
| 116 | Songping Village,Zhongzhai Town,Wenxian County,Longnan City,Gansu Province,China | 0.97 | high suitable area | HS |
| 117 | Jijiabao Village,Baoziba Town,Wenxian County,Longnan City,Gansu Province,China | 1.00 | high suitable area | HS |
| 118 | Baoziping Village,Baoziba Town,Wenxian County,Longnan City,Gansu Province,China | 1.00 | high suitable area | HS |
| 119 | Deshengqian Village,Baoziba Town,Wenxian County,Longnan City,Gansu Province,China | 1.00 | high suitable area | HS |
| 120 | Shangdanbao Village,Danbao Town,Wenxian County,Longnan City,Gansu Province,China | 0.78 | high suitable area | HS |
| 121 | Shangdanbao Village,Danbao Town,Wenxian County,Longnan City,Gansu Province,China | 0.93 | high suitable area | HS |
| 122 | Liziba Village,Tielou Tibetan Town,Wenxian County,Longnan City,Gansu Province,China | 0.84 | high suitable area | HS |
| 123 | Haxigou Village,Zhongzhai Town,Wenxian County,Longnan City,Gansu Province,China | 0.96 | high suitable area | HS |
| 124 | Zhongzhai Village,Zhongzhai Town,Wenxian County,Longnan City,Gansu Province,China | 0.96 | high suitable area | HS |
| 125 | Xigoushan Village,Koutouba Town,Wenxian County,Longnan City,Gansu Province,China | 0.85 | high suitable area | HS |
| 126 | Doujiawan Village,Koutouba Town,Wenxian County,Longnan City,Gansu Province,China | 0.67 | high suitable area | HS |
| 127 | Fengyuanshan Village,Shangde Town,Wenxian County,Longnan City,Gansu Province,China | 0.96 | high suitable area | HS |
| 128 | Tianchi Village,Tianchi Town,Wenxian County,Longnan City,Gansu Province,China | 0.97 | high suitable area | HS |
| 129 | Tianchi Village,Tianchi Town,Wenxian County,Longnan City,Gansu Province,China | 0.97 | high suitable area | HS |
| 130 | Tianchi Village,Tianchi Town,Wenxian County,Longnan City,Gansu Province,China | 0.97 | high suitable area | HS |
| 131 | Tianchi Village,Tianchi Town,Wenxian County,Longnan City,Gansu Province,China | 0.97 | high suitable area | HS |
| 132 | Zhangjiawan Village,Qiaotou Town,Wenxian County,Longnan City,Gansu Province,China | 0.86 | high suitable area | HS |
| 133 | Jijiabao Village,Baoziba Town,Wenxian County,Longnan City,Gansu Province,China | 1.00 | high suitable area | HS |
| 134 | Yangzhishan Village,Zhongzhai Town,Wenxian County,Longnan City,Gansu Province,China | 0.94 | high suitable area | HS |
